# Possible Causes of a Harbour Porpoise Mass Stranding in Danish Waters in 2005

**DOI:** 10.1371/journal.pone.0055553

**Published:** 2013-02-27

**Authors:** Andrew J. Wright, Marie Maar, Christian Mohn, Jacob Nabe-Nielsen, Ursula Siebert, Lasse Fast Jensen, Hans J. Baagøe, Jonas Teilmann

**Affiliations:** 1 Department of Bioscience, Aarhus University, Roskilde, Denmark; 2 Institute for Terrestrial and Aquatic Wildlife Research, University of Veterinary Medicine, Foundation, Hannover, Büsum, Germany; 3 Fisheries and Maritime Museum, Esbjerg V, Denmark; 4 Zoological Museum, The Natural History Museum of Denmark, University of Copenhagen, Copenhagen, Denmark; The Australian National University, Australia

## Abstract

An unprecedented 85 harbour porpoises stranded freshly dead along approximately 100 km of Danish coastline from 7–15 April, 2005. This total is considerably above the mean weekly stranding rate for the whole of Denmark, both for any time of year, 1.23 animals/week (ranging from 0 to 20 during 2003–2008, excluding April 2005), and specifically in April, 0.65 animals/week (0 to 4, same period). Bycatch was established as the cause of death for most of the individuals through typical indications of fisheries interactions, including net markings in the skin and around the flippers, and loss of tail flukes. Local fishermen confirmed unusually large porpoise bycatch in nets set for lumpfish (*Cyclopterus lumpus*) and the strandings were attributed to an early lumpfish season. However, lumpfish catches for 2005 were not unusual in terms of season onset, peak or total catch, when compared to 2003–2008. Consequently, human activity was combined with environmental factors and the variation in Danish fisheries landings (determined through a principal component analysis) in a two-part statistical model to assess the correlation of these factors with both the presence of fresh strandings and the numbers of strandings on the Danish west coast. The final statistical model (which was forward selected using Akaike information criterion; AIC) indicated that naval presence is correlated with higher rates of porpoise strandings, particularly in combination with certain fisheries, although it is not correlated with the actual presence of strandings. Military vessels from various countries were confirmed in the area from the 7th April, en route to the largest naval exercise in Danish waters to date (Loyal Mariner 2005, 11–28 April). Although sonar usage cannot be confirmed, it is likely that ships were testing various equipment prior to the main exercise. Thus naval activity cannot be ruled out as a possible contributing factor.

## Introduction

Over the years, a number of very disparate causal factors have been assigned to cetacean strandings. These include, but are not limited to: behavioural errors, such as failure of navigation related to the use of the Earth's geomagnetic field [1]; atmospheric-oceanic events, such as hurricanes [2]; compromised health status caused by infectious diseases or effects of anthropogenic activities [3]–[6]; and other issues of anthropogenic origin, such as contaminants loads [7]–[10]. More recently, growing evidence also implicates a more direct role of human activities (particularly military exercises) in causing cetacean strandings through exposure to noise ([11]–[13] and review [14]). For example, mass strandings in beaked whales have been suggested to result from either acoustic trauma [15] or behavioural responses [16]–[19] following exposure to navy sonar. Exposure to navy sonar has also been implicated in strandings of other species [14], including harbour porpoise (*Phocoena phocoena*) [20] and, most recently, common dolphin (*Delphinus delphis*) [21]. A wider suggestion is that the animals are following basic, innate behaviours during times of extreme stress responses, such as seeking the ancestral “safety” of land (see [22] pages 82–83 and [23]). In the majority of cases it is perhaps most likely that a combination of factors are involved [2],[24]–[26]. To that extent, lunar and solar cycles have also been noted to correlate with, and potentially influence, stranding rates [27]–[29]. Finally oceanographic currents and wind will also play a role in determining if a stranding will occur at all [30].In addition to being directly implicated in causing strandings, as mentioned for beaked whales above, behavioural responses to acoustic exposures have also been seen to more generally increase the risk of detrimental interactions with further human activities in other cetacean species. For example, North Atlantic right whales (*Eubalaena glacialis*) responded to novel alarm signals by coming near, but not actually to, the surface, placing them at the highest risk of being struck by ships [31].

Similarly, higher entanglement rates were reported for humpback whales (*Megaptera novaeangliae*) that were exposed to underwater explosions [32]. Although the mechanism was not identified, three possibilities were suggested by the authors. Firstly, the acoustic trauma associated with the explosions could have disorientated the whales. Secondly, the ability of the whales to detect the nets acoustically may have been compromised as a consequence of temporary threshold shifts in hearing. Finally, it is quite possible that the whales were responding behaviourally to the explosions.

With regards to harbour porpoise, bycatch is typically the most commonly declared cause for strandings [33]–[35], although disease, contaminants [8] and lethal interactions with bottlenose dolphin (*Tursiops truncatus*) [36] have also been receiving increased attention over the last decade or so. Bycaught animals may become stranded as many cetaceans are simply thrown back into the sea or fall out of the nets before being hauled on board. Set gillnets are especially problematic for this species [35]. However, while mass strandings (typically defined as two or more individuals stranding in the same location, but not a mother and calf) are not uncommon in some species (e.g., pilot whales; *Globicephala* spp.) they are rare for harbour porpoise [23]. Generally, only single animals will strand at any given time [33]. Accordingly, unusual mortality events (UME's) for porpoises are generally characterised by a substantial increase in the rate of strandings, rather than the presence of a typical mass stranding. For example, 15 porpoises that stranded in one month (compared with six per year) were declared to constitute an UME in Washington State, USA, in 2003 [20]. Similarly, 28 porpoises that stranded over a 2-month period in Sweden in 2007 were also considered to be a mass mortality event [37].

**Figure 1:**
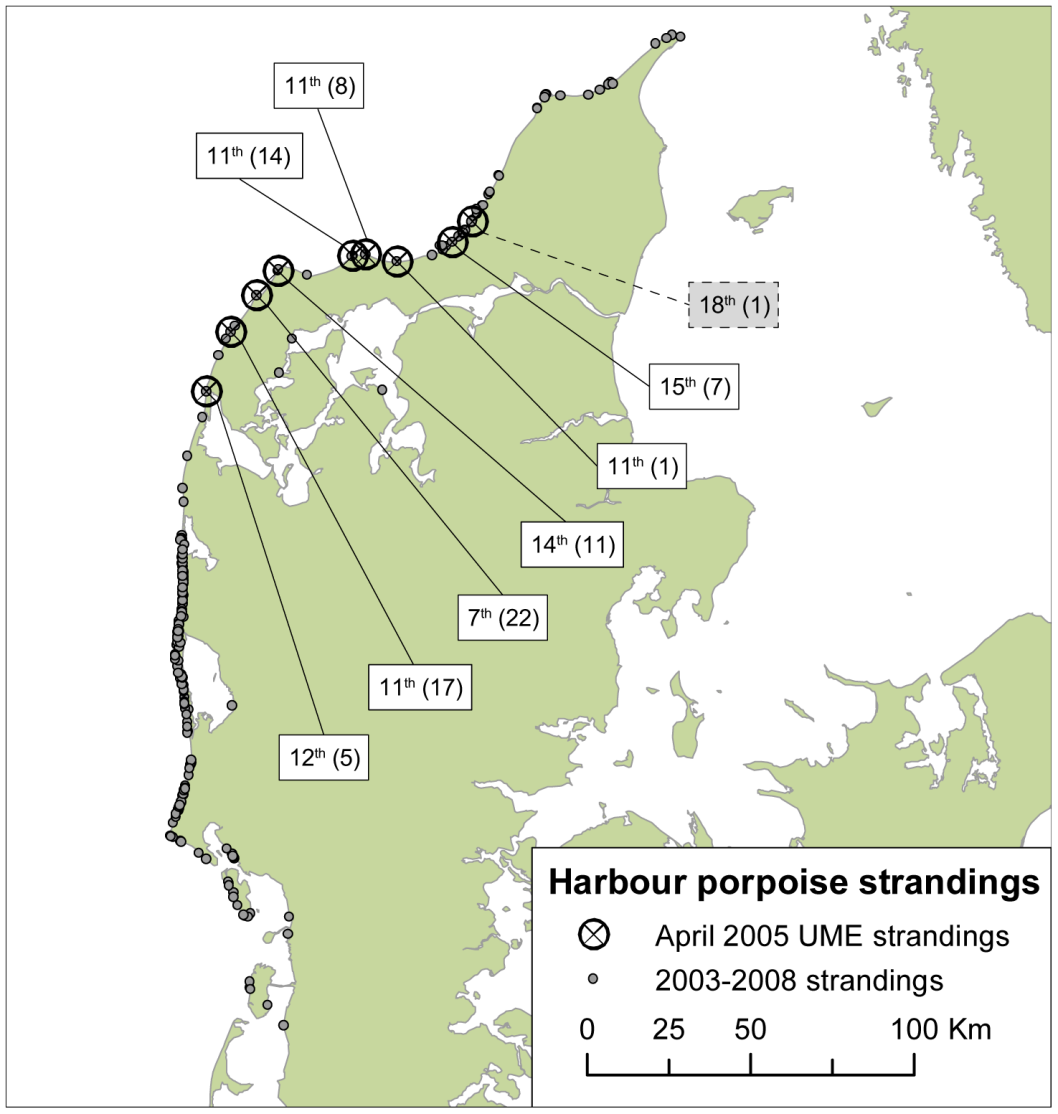
Locations of harbour porpoise strandings on the west coast of Denmark, 2003–2008 (N = 438). Circled crosses represent strandings in the April 2005 UME, with date (in April) given at each location, with the number of porpoises found in parenthesis. The location of the additional stranding on the 18^th^ is also noted.

**Figure 2:**
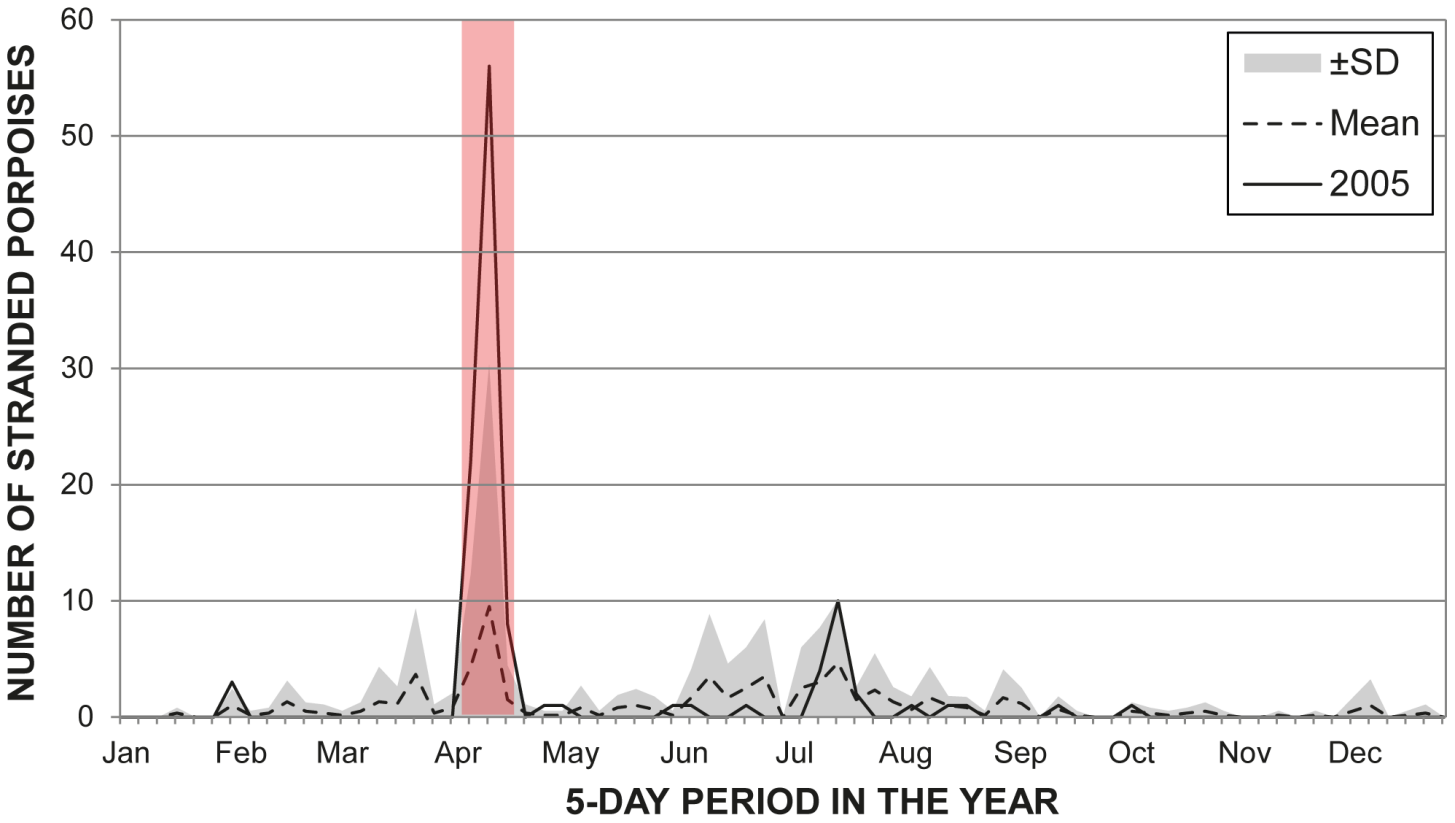
Harbour porpoise strandings on the Danish west coast, 2003–2008 (N = 438). The data is aggregated in 5-day periods and the time of the 2005 UME is highlighted in red. The mean line and standard deviation (SD) were calculated from values in the same 5-day periods across the entire 2003–2008 period, including 2005.

Over a period of just nine days between 7^th^ and 15^th^ April 2005, 85 porpoises stranded along approximately 100 km of coastline in Northwest Denmark (Figure 1). (It should be noted that an additional animal stranded in the same area on the 18^th^ April, but was not included in the analysis of the UME.) This unprecedented rate of strandings is substantially higher than the weekly mean stranding rate for the whole of Denmark during 2003–2008 (see Figure 2), both for any time of the year, 1.23 animals/week (ranging from 0 to 20 during 2003–2008, except April 2005), and specifically in April, 0.65 animals/week (0 to 4, same period). Also in April 2005, several lumpfish (*Cyclopterus lumpus*) fishermen confirmed that they had been catching unusually large numbers of porpoises in their nets and an early lumpfish season was consequently blamed. This level of strandings has not been repeated since in Danish waters, suggesting that the fishery itself could not be the lone reason for this UME. Instead, it is quite possible that one or more additional factors contributed to the UME, through cumulative or synergistic interactions that increased the risk of bycatch and/or entanglement.

The aim of this study was to investigate the potential role of the various factors that may have contributed to the observed UME in Danish waters during spring 2005, including anthropogenic noise. To achieve this, we produced a two-part statistical model of fresh porpoise strandings on the North-West coast of Denmark that incorporated not only sound-producing human activities and fish landings, but also various other possible factors. We also included consideration of various possible contributing elements that did not lend themselves to statistical analysis, such as post-mortem findings and wind action.

## Methods

Strandings data of dead marine mammals is a culmination of four types of factors, which can be very hard to distinguish between in the record [30]. These factors can influence: (1) abundance of animals present in the area; (2) mortality rate; (3) arrival of the carcass on the beach; and/or (4) probability of discovery. Although the probability of discovery may vary from district to district in Denmark due to accessibility and local coast patrolling procedures, we can consider that it remains reasonably constant through time in any particular district. Thus, in this study, we can limit the analysis to consideration of factors that can influence abundance, mortality rates or arrival of the carcass on the beach.

In summary, for factors influencing abundance we considered: season (e.g., migrations); and availability of prey (which itself may be dependent upon oceanographic features, such as fronts). With regards to mortality, we considered: season (e.g., breeding); fisheries activity; human activities known to produce noise; and environmental factors that have been shown or suggested as leading to strandings (lunar cycles, solar activity/cycles, variability in magnetic fields, extreme weather or oceanographic events, and also seaquakes). Finally, with regards to arrival of carcasses on the beach, we considered: wind direction and speed; and ocean currents (including tidal patterns) which could aid strandings mechanically. It should be noted that several factors can influence strandings data in more than one way.

The data needed for our retrospectively analysis were collected from numerous sources for the period 2003–2008. This period was selected as stranding data from prior to 2003 was not collected systematically or aggregated into a single database and as available modelled oceanographic data only extended up to and including 2008. The data was aggregated over 5-day periods to facilitate inclusion of the modelled oceanographic data that consists of 5-day mean values.

It was determined that a two-part statistical model would be created using R (version 2.14.1 [38]) of fresh porpoise strandings on the North and West coast of Jutland, Denmark, as this represented the most focused area incorporating the stranding site possible, given the fisheries landing data available (see below). The first part was a binomial linear model to investigate the occurrence (i.e., presence and absence) of fresh porpoise strandings and the second part was a Gaussian linear model with Gaussian error distribution that explored the correlation between the explanatory variables and the number of fresh porpoise strandings, when they were present.

### Strandings data

Data on 791 Danish harbour porpoise strandings was provided by the Danish Nature Agency, Ministry of Environment (the DNA). The Danish stranding network is operated jointly by the DNA in collaboration with the Fisheries and Maritime Museum and the Natural History Museum of Denmark. The strandings data is based on reports from the strandings network to the DNA from each local coastal district in Denmark. These districts may rely on official personnel in addition to reports from the public to monitor for strandings, but use official personnel only to collect data and occasionally also collect carcases. Registration of strandings with the DNA prior to 2003 is much less consistent and thus this data was excluded. However, the DNA reports that the effort has been reasonably constant since that time.

We decided to only include animals determined to be fresh-dead from the North Sea and Skagerrak coastlines in the analyses, to more accurately link the environmental variables in the stranding area to the date of the death of the porpoise. It was thus necessary to assign a ‘freshness’ value, based upon the noted condition of each individual in the stranding reports (fresh, not-fresh, and unknown). (Although freshness values are assigned to animals that are selected for full laboratory necropsies, it should be noted that the Danish Stranding Network does not yet use the European Cetacean Society decomposition condition code system, initially called simply “condition code”, that has become the standard throughout most of Europe [39].)

### Post mortem examinations

It was not possible to gather data retrospectively on the general health of the animals in the population. We were, however, able to review the available information on the pathological state of some of the individuals involved in the UME, although this could not be incorporated directly into the statistical analyses.

On-site examinations were carried out by DNA representatives and limited notes regarding condition were recorded. Detailed post-mortem examinations were performed as described by Siebert *et al.* [6] on 19 harbour porpoises that stranded on the 7^th^ April, 2005. All specimens, with the exception of one, were stored frozen at −20 C° from within 48 hours of discovery until examination. At post-mortem examination the state of preservation varied between fresh (state 2) and advanced decomposition (state 5) [39]. The nutritional status was judged on ten individuals. In some specimens internal organs were missing, permitting full examination of only eight animals.

Depending on the state of preservation the carcasses were examined for external lesions, in particular those characteristic of bycatch. These include net marks, as abrasion of fin or tail fluke or incision wounds in the abdominal wall, haemorrhage in the head region, including the eye chamber, and severe pulmonary oedema [6],[34],[39],[40].

All organ systems were examined macroscopically and samples of lesions and/or different organ systems were taken according to Siebert *et al.* [6]. Sections of 5 µm thickness were stained by hematoxylin and eosin and selected sections were stained by using Elastica *van Gieson* and Periodic acid Schiff stains (PAS) to further characterise lesions. In addition, lung, liver, kidney, spleen, intestine, intestinal lymph nodes and suspicious lesions were submitted for bacteriological examination [41].

### Environmental data

Environmental factors (including season) can influence strandings data in two main ways. Firstly, the porpoise distribution is known to be influenced by oceanographic fronts [42] and to have a seasonal component, probably based on a combination of prey availability and the underlying environmental variables [43]. We considered these proxies for porpoise distribution as, unfortunately, direct data on harbour porpoise densities was not available for the retrospective analyses. Similarly, no tags in the on-going Danish tagging program [43] were deployed at the time of the UME. Likewise, there was no data available on the distribution of potential competitors or predators, including other cetaceans and sharks. Secondly, environmental factors can also influence the likelihood of carcasses reaching the shore [30]. These include wind direction and speed, oceanographic currents, and tidal height. Meteorological data (including precipitation) was provided for all areas of Denmark by the Danish Meteorological Institute (DMI). Notably, this included precipitation data, which could be used as a proxy for extreme weather events, air temperature and received light. DMI also provided wind direction information at the three sites closest to the stranding area (Hanstholm, Thyborøn, and Hirtshals), and tidal height from the centre of the stranding area (Hanstholm). Finally, DMI also provided oceanographic data (including temperature) for both bottom and surface waters though the 3D circulation model DMI-BSHcmod (see [44]) for the period 2003 to 2008. This model has previously been validated against actual observations [44]. The modelled oceanographic data was provided in the form of mean values over 5-day periods with a horizontal resolution of 6 nautical miles over the entire area of interest. The vertical grid consists of up to 50 depth layers of variable thickness. (These are 8 m in the surface layer, 2 m in the next 36 layers and gradually increasing from 2 m to 155 m in the remaining layers towards the bottom. Water depth in the Skagerrak varies from 8 m to 666 m with the maximum depth in the Norwegian Trench.) The modelled oceanographic data included North-South and East-West flow components, temperature and salinity.

While it is possible that winds moving onshore from the west or north could have enhanced (if not caused) the 2005 UME by helping to push floating carcases onto beaches, wind direction could not be easily aggregated over the 5-day periods. It was thus not possible to include wind direction in the statistical analysis. Without this information it was meaningless to include wind speed into the model. However, both were considered outside the model with regards to the 2005 UME.

**Figure 3:**
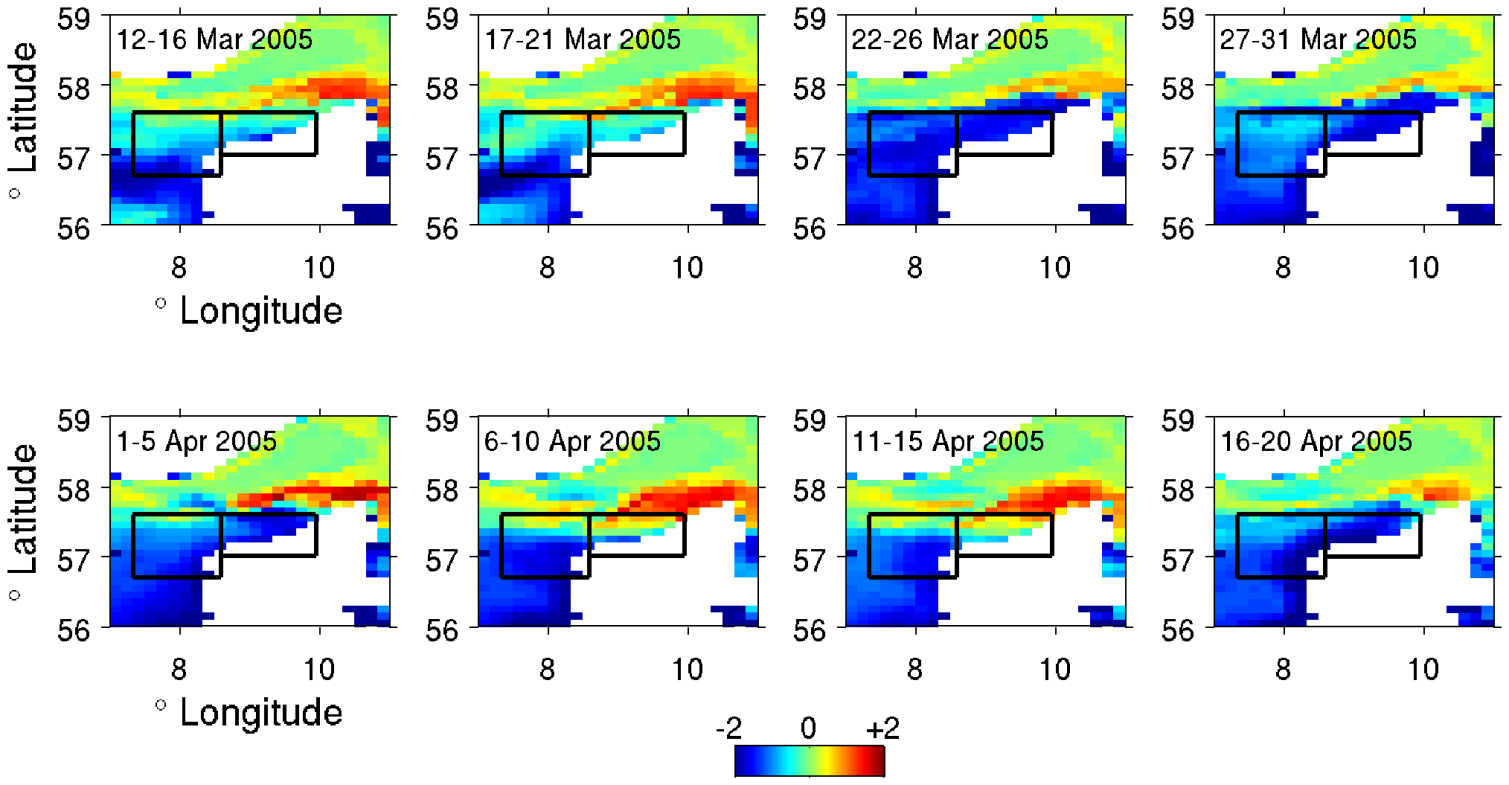
Temperature anomalies of Skagerrak and Danish North Sea bottom waters in March and April 2005. Each image represents the difference in DMI-BSHcmod modelled bottom temperatures across 5-day periods relative to the 2003–2008 mean bottom temperatures from the same periods. Each pixel represents one 6 nm^2^ model area. Note the movement of unusually warm bottom waters into the stranding area between 6^th^ and 15^th^ April. Black rectangles indicate the location of the Western and Eastern box respectively.

Likewise, it was clear that we could not include all of the meteorological and oceanographic variables into the model, due to collinearity. Thus the DMI tidal data were included after being combined into a single variable, tidal range, which was calculated as the difference between the greatest and lowest tidal height over the 5-day period. Similarly, we chose to use two compound variables based upon the DMI-BSHcmod data to reflect oceanographic conditions that we assumed most relevant for the porpoises. These were: (1) a 25-day (five 5-day periods) running mean in the temperature of bottom water in the western part of the stranding area (hereafter referred to as “BW Temp”; see Figure 3); and (2) the relative temperature difference between the bottom waters of the western and eastern part of the stranding area (hereafter referred to as “W-E Temp”; see Figure 3). The former, BW Temp, was dominated by seasonal variation and was thus retained to act primarily as a proxy for seasonal changes in both environmental values and also porpoise geographical distribution in the absence of actual distribution data. Likewise, this will also embody any seasonal signal relating to porpoise breeding cycles. The latter, W-E Temp, was retained to reflect the presence of oceanographic frontal systems and any other unusual oceanographic features in the area.

Similarly, to avoid collinearity within the meteorological data, only precipitation was included, which was deemed to be relevant as the authors have observed porpoises held at facilities reacting strongly to rain. This variable was also deemed likely to be the best indicator of extreme weather events.

Other considerations included lunar cycles, solar activity, magnetic field observations and seaquakes. Lunar cycles were dismissed quickly as this data would obviously be correlated with tidal range data. Solar activity data was also considered and ultimately excluded from the analyses for various reasons. Most notably, higher stranding rates in sperm whales have been associated with shorter solar cycles only over large spatial and temporal scales [27]. Additionally, 2005 was close to the minima of a longer than normal solar cycle [45]. Solar-driven geomagnetic anomalies have also been seen to correlate with sperm whale strandings across the North Sea [28]. Here the available data on geomagnetic activity included aa-index data (which is a simple global geomagnetic activity) from the British Geological Survey – BGS – Geomagnetism program (http://www.geomag.bgs.ac.uk/) or direct magnetic field observations that were available from the Brorfelde Observation Station (National Space Institute at the Technical University of Denmark – DTU, but held at the World Data Centre: http://www.space.dtu.dk/english/research/scientific_data_and_models/magnetic_ground_stations.aspx#map). However, the first was a global index and the latter was data from a site located over 200 km southeast of the stranding area, on the far side of Denmark. (Another DTU site, Rømø, located nearly 200 km almost due south of the stranding area did not start recording data until September 2005.)

We excluded these geomagnetic data from our model for the following three reasons: (1) we determined that neither of these data sets would be representative of the specific local situation of the strandings site; (2) the global or distant nature of the data would likely have caused a wider-ranging UME, if these were to have been acting as contributing factors; and (3) we were not convinced of the importance of magnetic fields to harbour porpoises, which are a highly coastal species that likely have a range of other cues available to them for navigation.

Finally, a search for earthquakes in Denmark, the Kattegat, Skagerrak and the contiguous Danish and Norwegian North Sea in the data held at International Seismological Centre [46] over the three weeks prior to the 2005 UME (search dates 17^th^ March 2005 to 8^th^ April 2005) revealed only five shallow minor events (likely magnitude 2.6 or below) on or near distant parts of the Norwegian or Swedish coastline. This is in comparison to 21 minor events (up to around a magnitude of about 3.4), include one approximately 10 km away from the northernmost tip of Denmark in the Kattegat over the same period in 2004. Furthermore, although the various local districts are only required to report stranding figures once a year, early indications are that August and September 2012 were unremarkable in terms of the number of porpoise strandings that occurred, despite the widely reported unusually shallow magnitude 4.1 earthquake that occurred near Anholt in the Kattegat Sea on 6^th^ August 2012. Given these facts and that there were no seismic events at or just prior to the 2005 UME, we decided to also exclude this data from the analysis to minimise the number of variables.

### Fishing data

The Danish Directorate of Fisheries, Ministry of Food, Agriculture and Fisheries provided the Danish daily landing weights per species for all fish caught throughout the period for both the Skagerrak and the Central North Sea International Council for the Exploration of the Sea (ICES) areas. They also provided an indication of proportional use of gear for the various fisheries over the entire period, although this was limited to catches over 1000 kg only. Thus, gear usage and changes could not be considered in a quantitative manner in statistical analyses, although the role of different gears could still be considered more qualitatively in light of the results.

Although gillnets are the predominant known cause of porpoise bycatch [35], trawls are known to be problematic for other species [47]. Given that we were seeking possible causes for a highly unusual stranding event, we included these fisheries as well. Additionally, the landings data do not simply represent an indication of bycatch threat, but also the presence of potential prey species, an indication of productive areas and potentially the presence of fine-scale oceanographic features as well. However, to reduce the variables, any fin- or shellfish species caught predominantly using pots or boat dredges were excluded from the analyses as these fishing methods are thought to pose virtually no risk to porpoises and the species targeted are not thought to be prey items. Similarly species with mean annual landings (per area) of less than 100,000 kg were removed.

The remaining catch data, representing 88 species-ICES area combinations (e.g., lumpfish caught in the Central North Sea: ‘N Lumpfish’), were then subjected to a principal component analysis (PCA) to further reduce the number of variables to be included into the statistical model. This allowed us to avoid including redundant variables in the model and to avoid the problems that arise as a consequence of multi-collinearity in statistical models. We determined that it would be necessary to reduce the influence of isolated zero values in the 5-day catch totals on the PCA. These could have resulted from mis-reporting, landings reported the first day of the next period, or some other artificial factor, rather than representing an actual lack of fish presence or fishing activity in a particular period. To achieve this, we replaced zero values with the running mean over 25 days (five 5-day periods). This process still retained sequential zero values that reflect seasonal takes (due to regulation or fish absence). Finally, given the large number of potential fishing variables, we decided to retain only as many PCA factors as needed to describe 50% of the variation in the catch data, as we anticipated that inclusion of more variables would make the model too large and impracticable (see discussion of axis selection in [48]). The ninth PCA axis brought the cumulative variation to just over 50%, so the first nine axes were included in the final model selection process.

### Data on other human activities

Although no data on chemical pollutants could be found, information on other possible anthropogenic factors was obtained. Noise is known to have a variety of impacts on marine mammals [49]–[52] [49–52]. Thus we decided to focus on noise-producing activities as a proxy for noise exposure, as actual noise data was also not available. However, the available data on human activities was generally more limited than other data sets due to concerns over proprietary data, confidentiality, or national security. Given the largely unrecorded nature of leisure activities, such as the use of speed boats or smaller commercial fishing vessels (which was, in any case, likely to have a seasonal signal associated with the environmental data mentioned above), we decided to focus on previously identified ‘commercial level’ activities. These were: seismic surveys for oil and gas exploration; pile-driving for construction of wind-farms and other coastal and offshore developments; commercial shipping; and military activity.

The Danish Energy Authority provided information on seismic survey activity in Danish waters for the stranding period. Similarly, information was obtained from the website of 4C Offshore Limited (http://www.4coffshore.com/) regarding construction periods of offshore wind farms (and thus periods of pile-driving) from 2003 to 2008. Unfortunately, detailed shipping data could not have been included as it was not available (e.g., land-based AIS tracking was not fully implemented in Denmark until the summer of 2005). Finally, information about military activities in Danish waters from 2003 to 2008 was provided by the Danish Navy and all this was included in the analysis (see Table S1). One particular exercise, the NATO exercise Loyal Mariner 2005 (LM05), was the largest to date in Danish waters and ran from 11^th^ to 28^th^ April, 2005. (The source for these dates was: http://forsvaret.dk/LoyalMariner05/eng/Pages/default.aspx. Last accessed 29th May 2012. It should be noted that the available information, even among official Danish Navy & NATO documents, is conflicting, with some sources reporting the closing date to be 29^th^ April.) Additional information on this exercise was provided by various other navies through official and unofficial requests (see Table S2).

There were no wind farm-related pile-driving in Danish waters at any point in 2005 (4 C Offshore Limited; http://www.4coffshore.com/). Similarly, the only seismic activity in Danish waters during the first two weeks of April, 2005 was part of a longer survey from 7^th^ March to 24^th^ September 2005, which did not come within 200 km of the stranding area at any point during the entire survey period (pers. comms., Danish Energy Authority). We thus discounted oil and gas-related activity as being unlikely to have been a factor in the comparatively brief 2005 UME, although the sound energy from these surveys may have been present in the area throughout the entire summer of 2005. For example, sound from seismic surveys from coastal waters of USA has been detected on the Mid-Atlantic ridge [53] although the local waters of Denmark and the North Sea are much more shallow, which will almost certainly limit propagation [54]. Consequently, the only sound-producing activity that could be included was military activity.

With regards to this activity, investigations into LM05 found that many ships had arrived in Danish waters some time before the exercise (see S2). Pre-exercise manoeuvres and testing prior to the main event were also reported. Such mini-exercises, last-minute training and equipment testing are not uncommon (pers. comm. Michael Jasny, National Resource Defense Council). For example, according to information acquired from the British Royal Navy, five ships (British and Canadian) were known to be moving through the stranding area conducting training not involving sonar from the evening of 7^th^ April. Unfortunately, the precise whereabouts of the 80 other ships remain almost completely unaccounted for (see S2 for details of the responses of the various navies to enquiries). However, a number of vessels are likely to have been at least transiting through the UME area on the 7^th^ April as the Defence Command Denmark (Danish Defence) report the arrival of many of the ships in Frederikshavn on the 8^th^ April, with more arriving over the following couple of days and others docking in Bergen, Norway around that time (e.g., http://forsvaret.dk/LoyalMariner05/eng/News/Pages/default.aspx. Last accessed 29^th^ May 2012).

Based upon the information available on LM05 the military activity was split into two categories: presence; and no known presence. The pre-exercise “present” period for LM05 spanned two 5-day periods. A similar pre-exercise period was also assigned to the other known military exercises in Danish waters between 2003 and 2008 to avoid statistical biases for the LM05 pre-exercise period, as the same level of detail was not sought (or available) for these other exercises.

### Model construction

All the environmental variables, with the exception W-E Temp, were logarithmically transformed. The resulting explanatory variables were thus: nine PCA axes representing over 50% of the variability in fishing landings; naval activity in Danish waters; W-E Temp; and the logarithmically transformed BW Temp, tidal range and precipitation. These four environmental variables were tested for collinearity. All pairwise correlations were found to be very weak (abs(r)<0.11) except between tidal range and both BW Temp (r = −0.25) and precipitation (r = 0.34). Despite these correlations, we kept these variables in the model as they represent disparate data and eliminating independent variables just to prevent multicollinearity would fundamentally alter the tested hypothesis [55]. However, the implications of the collinearity are discussed. The dependent variable in the first binomial part of the model, which was constructed to determine the relationship between the presence of fresh stranded porpoises and the independent variables, was a true-false indicator of stranding presence in the area of interest. Like many of the independent variables, the dependent variable in the linear part of the model, number of fresh harbour porpoise strandings, was also logarithmically transformed. As porpoise strandings in Danish waters generally involve only a few individuals in sporadic events, the occurrence and number of strandings in each 5-day period are thus independent. This second model included only 5-day periods where fresh stranded porpoises were present.

The process of model construction was identical for each part of the model. The initial model included the appropriate dependent variable (binomial presence-absence or the log-transformed number of strandings when present) and all the independent variables. Interaction terms were then added in turn by forward selecting the interaction that offered the greatest reduction in Akaike information criterion (AIC). The stopping criteria was determined to be the point where adding further interaction terms ceased to improve the model (i.e., reduced the AIC value) by at least 2 points. The interaction terms that were assessed for incorporation into the model were those between the anthropogenic term, navy presence, and each of the other independent variables.

Additionally, prior to the forward selection of interactions, it was necessary to add the different independent variables in the initial model in turn to assess their relative merits. This was done through a process similar to that of forward selection from a null-model, by selecting the variable that offered the greatest reduction (or smallest increase) in AIC. The stopping point for this process was the creation of the full initial model (i.e., the inclusion of all the independent variables). This did not form part of the forward selection process itself, but was only undertaken to assess the contributions of the independent variables to the initial model.

Accordingly, this model construction was statistically testing factors against two null hypotheses. Firstly, that none of the explanatory variables included were correlated with the presence of strandings over 5-day periods on the North and West coast of Jutland, Denmark. Secondly, that none of the explanatory variables included were correlated with the number of strandings (i.e., the rate of strandings) when they were present in a 5-day period on the North and West coast of Jutland, Denmark.

## Results

### Post-mortem findings of harbour porpoises

Of the 85 animals in the UME, signs of bycatch were reported on site in 27 porpoises (e.g., missing extremities, net marks, etc.), with bycatch specifically noted as the cause of death in six individuals. This represents nearly one third of the UME animals and can be compared with 103 out of 706 of the remaining porpoises in the 2003–2008 Denmark-wide stranding record reported to have similar signs of bycatch.

With regards to the detailed post-mortem investigations, the nutritional status was judged in 10 of the 19 porpoises dissected in 2005. Three individuals were found to be in a good nutritional status, six in a moderate status, and one animal was emaciated. Six of the eight individuals subjected to a full necropsy displayed mild to severe infection by nematodes in the bronchial tree and blood vessels. Mild to severe bronchopneumonia was found in six specimens. Pulmonary edema was diagnosed in eight, pulmonary congestion in four cases and pulmonary emphysema in three cases. Thrombosis and thrombarteritis, periarteritis and nematodes in the right heart chamber were only found in single cases. In another case, nematodes were found in the first stomach compartment. Ulcerative and granulomatous gastritis and catarrhal enteritis was found in one case each and trematodes were found in the liver in four cases. One pregnant female was diagnosed as having died intra partum due to a suppurative-fibrous peritonitis with ascitis and pyometra. Five specimens showed mild to severe parasitic infection in the ear sinuses. Net-marks or cut parts of flukes indicative of bycatch were found in six porpoises. Bacterial examination revealed alpha- and beta-haemolytic *streptococci*, *Clostridium perfringens* and *Escherichia coli* associated with inflammatory lesions.

Six of the 19 porpoises were delivered with by-catch reported as cause of death, conclusions that were supported by the pathological investigations. Three additional specimens were suspicious for by-catch based on the pathological findings. Two of those individuals also suffered from a moderate or severe bronchopneumonia. Two further porpoises were found to have died as a result of bronchopneumonia, one of bronchopneumonia and hepatitis, one of dystocia with final streptococci septicemia. The cause of death of the remaining eight animals remained unclear, possibly as a result of the state of decomposition. There was no evidence of morbillivirus, herpesvirus, *Toxoplasma gondii*, *Brucella* spp. or algal biotoxins found in any of the specimens. Similarly, no histological findings clearly indicative of acute intoxication by organic or inorganic pollutants were found.

Lesions have previously been found in deeper-diving cetaceans stranded after exposure to some military sonars, potentially relating to gas and fat emboli similar to decompression sickness in humans [11],[12],[56]. Such lesions were not found in the above-mentioned porpoise specimens, although the diagnosis of those lesions is very limited due to the state of preservation of the carcasses. There were also no gas-filled fibrous cavities in the liver as described elsewhere [11],[12].

### Fishing activity and the PCA

**Figure 4:**
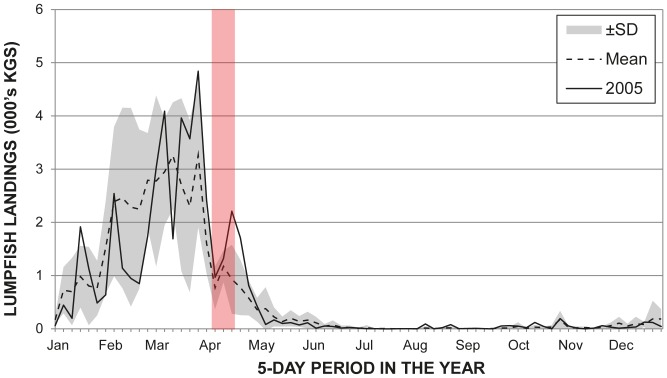
Lumpfish landings from the Skagerrak and Central North Sea, 2003–2008. The data is aggregated in 5-day periods and the time of the 2005 UME is highlighted in red. The mean line and standard deviation (SD) were calculated from values in the same 5-day periods across the entire 2003–2008 period, including 2005.

Although the initial comparison of the lumpfish landing data across the years 2003-2008 revealed an unusually high landing of lumpfish at the end of the UME period in 2005 (in comparison to the other years, but not in terms of the overall numbers), it did not begin until the UME was in its final stages (see Figure 4). Furthermore, 2005 was not an unusual lumpfish season in terms of either season onset or peak landings. Despite this, the variation present in the landings data for this species was still included in the full statistical analyses through the axes produced by the PCA. The variation in lumpfish landings formed major constituents of all the variation in all the landings data captured by the first and second principal component axes (PC1 and PC2; full details of the PCA results are included in Table S3 and Figure S1). PC1 also incorporated a reasonable amount of variation in the Atlantic cod (*Gadus morhua*) landings in both regions, and the turbot (*Scophthalmus maximus*) landings in the Skagerrak, in addition to that of numerous other minor fisheries, totalling 20% of all the variability in the landings data. PC2 incorporates another 9% of all the variation in the landings data. PC3 to PC9 respectively contributed between 5.6 and 1.9% of the total variance in landing data to the cumulative total variance to reach the 50% target.

Of note with regards to important prey species in the region (based on stomach contents [57]), the variation in the landings data for Atlantic cod was somewhat spread over all nine PCs, although much was contained within PC1, PC2 and PC3. These PC axes also clearly incorporated the majority of the variance seen in the Atlantic herring (*Clupea harengus*) landings data. Finally, PC3 and, to a lesser extent, PC1 captured much of the variance in the less important whiting (*Merlangius merlangus*), with Skagerrak landings in particular a primary constituent of PC3. With regards to important fisheries for bycatch (see [35]), in addition to the distribution of cod mentioned above, turbot landings from the North Sea were strong components of PC3, PC8, and PC9, while turbot landings from the Skagerrak were another major component of PC1, but also featured heavily in PC4. Of less importance for bycatch (see [35]), a large majority of the variation in European hake (*Merluccius merluccius*) landings was also incorporated into PC1, while European plaice (*Pleuronectes platessa*) landings are not very well represented by any single PC axis.

### Final statistical models

**Figure 5:**
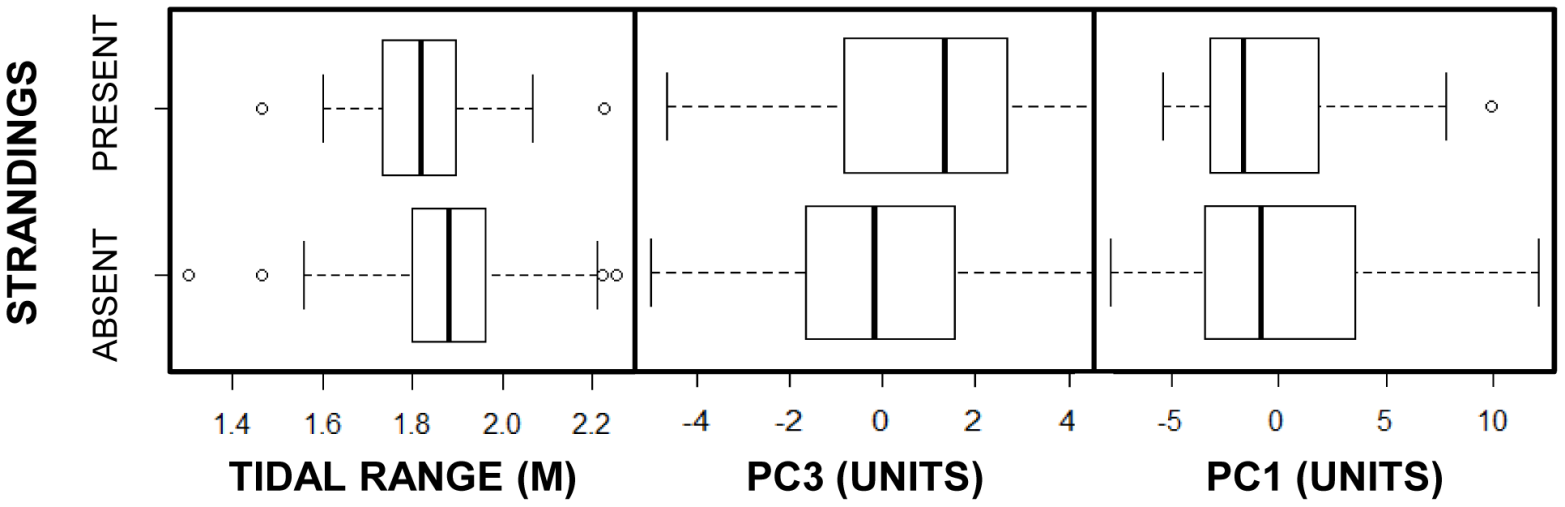
Important model factors for the occurrence of Danish west coast fresh porpoise strandings, 2003–2008. Identified by AIC, these factors were tidal range (m), PC3, and PC1 (in order added into the model). The bold lines represent median values, the boxes are 25^th^ to 75^th^ percentiles and the whiskers extend to the most extreme data within 1.5 IQR (interquartile range) of these lower and upper quartiles. Outliers are represented by points. PC1 & PC3 are products of the PCA and are, thus, unit-less.

During the construction of the full binomial model for presence of strandings (see Table 1), PC3 and PC1 were the only 2 additions that improved the model (i.e., reduced AIC by 2 or more) when they were added. In addition, tidal range was the first term added, even though this increased AIC, as it did so by a smaller amount than the other terms. No interaction terms were found to improve the model through forward selection (see Table 1). The presence of strandings was thus associated with slightly smaller tidal range and potentially also a marginally lower value of PC1 and a higher PC3 value (see Figure 5).

**Table 1:** The relative importance of independent variables in the final binomial model.

Constant for:	Sum Squ's	AIC
**Tidal Range**	**0.58943**	**325.1943**
**PC3**	**1.11772**	**320.4170**
PC7	0.39522	318.7963
PC2	0.64374	316.8681
PC8	0.22837	316.5625
PC9	0.15620	316.8523
BW Temp	1.08001	317.3671
**PC1**	**0.08162**	**311.5515**
W-E Temp	0.18757	311.7329
Precip	0.14781	311.8773
PC4	0.06913	312.4979
PC6	0.11684	314.0380
PC5	0.03095	316.0092
Navy	0.00691	317.9917
No interactions included		
Residuals	43.85546	na

The sum of squares for each variable are reported for the final model. The variables were added one at a time until all were included, using the lowest AIC so that the best model was maintained at all times. Variables that reduced the model by at least 2 AIC (in addition to the first variable added) are highlighted in bold. No interactions were found to improve the full model through forward selection. Each variable has one degree of freedom. The final model had residual 415 degrees of freedom. BW Temp is the 25-day (five 5-day periods) running mean in the bottom temperature of the Western box (see Figure 3). W-E Temp is the difference in the temperature of the bottom waters between the Western and Easter boxes (see Figure 3). Navy is the presence-absence of naval activity, with “No Known Activity” as the base category.

**Figure 6:**
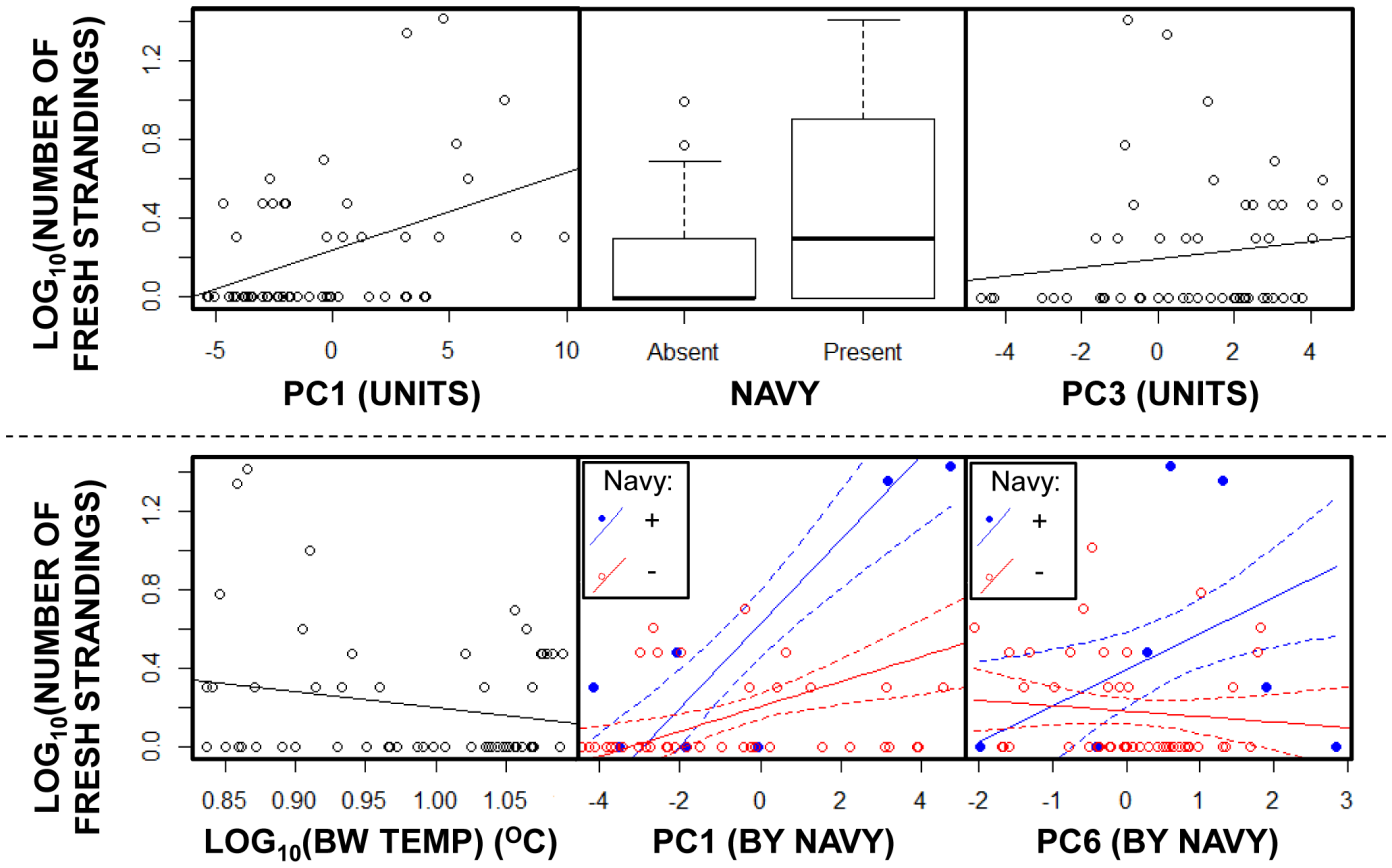
Important model factors for the rate of Danish west coast fresh porpoise strandings, 2003–2008. Specifically, Log_10_ fresh harbour porpoise strandings correlated with PC1, navy presence, PC3, and BW Temp (log °C), as well as the interactions between Navy presence and both PC1 and PC6 (in order of inclusion into the model). For BW Temp, PC1, and PC3 the data is presented with linear regression lines; for the navy presence, the bold lines represent median values, the boxes are 25^th^ to 75^th^ percentiles and the whiskers extend to the most extreme data within 1.5 IQR (interquartile range) of these lower and upper quartiles. Outliers are represented by points. For PC1 and PC3 by navy state, the data is presented with linear regression lines (continuous) and 95% confidence intervals (dotted) separately for periods where the navy was present (blue) and absent (red). PC1, PC3, and PC6 are products of the PCA and are, thus, unit-less.

With regards to the number of strandings once they were present, PC1 (the first term added), Navy presence, PC3, and BW Temp all improved the model when added (see Table 2). Increases in PC1 and PC3 were correlated with a higher rate of strandings, as was the presence of naval activity, although a higher running average of bottom water temperature in the area was correlated with a lower rate of strandings (see Figure 6). With regards to the forward selection after all the single terms had been added to the starting model, interactions between naval presence with both PC1 and PC6 were found to improve the model (see Table 2). Navy presence increased the positive correlation of both axes with the number of strandings (see Figure 6).

**Table 2:** The relative importance of independent variables in the final linear model.

Constant for:	ΔR^2^	Sum Squ's	AIC
**PC1**	**0.1804**	**1.134820**	**31.33793**
**Navy**	**0.1081**	**0.456196**	**25.42109**
**PC3**	**0.0532**	**0.360925**	**23.06614**
PC8	0.0306	0.246615	22.40061
PC2	0.0265	0.070388	21.98523
**BW Temp**	**0.0555**	**0.393988**	**18.56037**
Precip	0.0278	0.401684	17.63678
PC5	0.0213	0.084563	17.27966
PC9	0.0172	0.037726	17.30867
PC7	0.0096	0.139504	18.17626
PC4	0.0009	0.005242	20.06849
Tidal Range	0.0006	0.004691	21.99654
W-E Temp	0.0001	0.008105	23.98344
PC6	>0.0001	0.000609	25.98015
**PC1 x Navy**	**0.1123**	**0.705880**	**12.63238**
**PC6 x Navy**	**0.0663**	**0.417054**	**3.087888**
Residuals	0.2896	0.216124	na

The sum of squares and ΔR^2^ for each variable are reported for the final model. The variables were added one at a time until all were included, using the lowest AIC so that the best model was maintained at all times. Variables that reduced the model by at least 2 AIC (in addition to the first variable added) are highlighted in bold. Interactions (between the lines) that reduced the model by at least 2 AIC were then added in turn in the same way through forward selection. Each term (variable or interaction) has one degree of freedom. BW Temp is the 25-day (five 5-day periods) running mean in the bottom temperature of the Western box (see Figure 3). W-E Temp is the difference in the temperature of the bottom waters between the Western and Easter boxes (see Figure 3). Navy is the presence-absence of naval activity, with “No Known Activity” as the base category. The F-statistic in the final model is 5.978 on 16 (variable) and 39 (residual) degrees of freedom (p = 2.591e-06).

## Discussion

A number of factors appear to have been involved in the strandings on the West Coast of Denmark in the period 2003 to 2008. In summary, the statistical analysis indicated that the variation in the presence of strandings may have been weakly influenced by tidal range (negatively), PC1 (negatively), which was dominated by the variation found in the lumpfish fishery, and PC3 (positively), which incorporated variation primarily from whiting landings in the Skagerrak. Similarly, the analysis revealed that the number of strandings was correlated with the variation contained within PC1 (positively), PC3 (positively), and BW Temp (negatively). There was also a positive association between the presence of the navy and a greater number of strandings, especially in combination with PC1 and PC6. Although PC6 included no especially dominating fishery elements, this axis did incorporate a reasonable proportion of the variation in hearing landings across both ICES areas. Finally, post-mortem analyses indicate that the suite of pathologies found in the UME animals is not unusual when compared to that of others stranded at other times and elsewhere in the North Sea (see below for details).

### Environmental factors

Tidal range appeared to be somewhat linked to the occurrence of fresh strandings along the Danish West coast. This is perhaps not surprising as it is likely to be mechanically involved in the arrival of carcasses on the beach. However, what was unexpected is that strandings may be more likely to be present when tidal range is lower. This finding may be due to a reduced likelihood of carcasses being washed back out to sea after stranding. However, we have to remember that tidal range was somewhat correlated with BW Temp (negatively) and precipitation (positively). Thus it is possible that these influences may have also indirectly played a role. In any case, there was no real relationship between the final model and the occurrence of strandings in the area, given the lack of reduction in AIC in the initial step of the forward selection process to construct the full statistical model (see Table 1).

With regards to the rate of strandings, when present, a lower BW Temp was somewhat related to a higher number of strandings. It is doubtful that a lower temperature in the bottom waters would represent a substantial thermal stressor for porpoises as they are exposed to a wide range of temperatures throughout the year and are thus unlikely to be affected by minor short-term changes. However, it is possible that this may have influenced porpoises indirectly through altering the distribution of either prey species or fisheries with high rates of bycatch. Such a relationship may have introduced a certain amount of collinearity into the statistical model and explain the change in the improvement of the binomial model offered by PC1 immediately after BW Temp was added to that model (see Table 1). One other alternative is that the relationship between BW Temp and stranding rate merely represents seasonal changes in either the distribution or the reproductive cycle of the Danish harbour porpoise. Although the seasonal distributions of North Sea porpoises are not known, the distributions of porpoises tagged within inner Danish waters vary seasonally, with more venturing into the Skagerrak and North Sea in autumn and winter [43]. Finally, the presence of a negative correlation between BW Temp and tidal range may also indirectly indicate an influence of increased tidal action in bringing dead animals to the beach due to the afore-mentioned collinearity between these two variables.

### Fishing

The role of fishing in the occurrence and number of fresh porpoise strandings in Danish waters is weakly supported by the model. Specifically, the variability in PC1, dominated by the lumpfish fishery, appears to be correlated with both occurrence (weakly and negatively) and number (positively) of strandings (see Tables 1 & 2; Figures 5 & 6). Furthermore, the fishing variation in PC3 was positively correlated with both stranding occurrence (weakly) and numbers (see Tables 1 & 2; Figures 5 & 6). The variation in landings data in PC3 is dominated by whiting in the Skagerrak (see Table S3). Consequently, the importance of this PC axis in both parts of the statistical model suggests a link between the presence of this lesser prey species (see [57]) and the occurrence and number of fresh strandings. This finding may indicate that greater number of fresh strandings occur simply when porpoise density is higher due to the presence of their prey, or it may simply reflect some untested combination of environmental factors that influence both predator and prey. In addition to these, the model selection process identified further positive relationships between both PC1 and PC6, in interaction with naval activity, and the number of fresh strandings (see below).

### Sound-producing human activity

Due to data availability and/or lack of presence, the only noise-producing activity that was incorporated into the model, military activity, could only be included in a binary way (major activity believed present or absent). Perhaps most important to note is that the binomial statistical model found no correlation between navy presence and the occurrence (presence verses absence) of strandings, which means that navy presence alone is not linked to fresh harbour porpoise strandings. However, naval presence does appear to be highly related to the number of strandings when they do occur. Both alone and in interaction with PC1 and PC6, naval presence in Danish waters is correlated with the number of fresh strandings, when they do occur. These interactions suggest that naval presence enhances the effects of the presence of the lumpfish (through PC1) and potentially also herring in the North Sea (through PC6).

### The 2005 Danish porpoise UME

#### Pathological factors

Harbour porpoises bycaught in the North Sea are rarely delivered directly by fishermen to the authorities. Thus, bycaught specimens must be identified as such when they strand (in rather high numbers [35]), which is a very difficult task in forensic pathology. With progressive decomposition of the carcass the identification of net marks and other bycatch-related lesions becomes more difficult. Furthermore, some net types do not produce net marks. Finally, as pathological investigations on bycaught harbour porpoises have shown, infectious diseases can be widespread among those animals, which also increases the difficulty of identifying bycatch among strandings [6],[58].

Systematic pathological investigations on stranded animals from the same stretch of coastline as the 2005 UME area are, unfortunately, unavailable from other years. However, the condition of the porpoises subject to detailed investigations elsewhere suggests that the above-mentioned suite of pathologies found in the UME animals is not unusual. Pathological findings (e.g. bronchopneumonia, gastritis, enteritis, hepatitis) in the UME porpoises were similar to those previously described for harbour porpoises from other areas of the North Sea [6],[40],[59]–[61]. Beta-haemolytic *streptococci*, *Clostridium perfringens*, and *Escherichia coli* were also previously isolated from harbour porpoises of the same subpopulation in the North Sea [6],[41],[62].

Perhaps it is instead more useful to consider the proportion of stranded animals noted by those undertaking the field assessments to have injuries typical of bycatch. The incidence of potentially bycatch-related injuries was much higher for the UME animals than is generally reported in the Danish record, suggesting that it was a major contributor to the enhanced rate of strandings for the period.

#### Other untested factors

With specific regards to the 2005 Danish porpoise UME, it seems unlikely that winds moving onshore from the west or north could have contributed, even if they were not integrated into the analyses. Wind direction at Hanstholm, in the middle of the stranding area, during and just prior to the UME was initially from the south, before switching to be more from the west-south-west on the 8th and 9th April, 2005. For the majority of the 10th through to the 13th April the wind was from the south-south-west, before switching to an easterly direction until well after the last stranding of the UME. Given that wind in the UME area was moving offshore at the time of the strandings, it seems unlikely that wind could have been a contributing factor.

Similarly, it seems unlikely that either wind farm-related pile-driving or seismic surveys for oil and gas exploration could have been major factors in the UME. Despite a lack of data, shipping was also determined to be an unlikely candidate as it is ever-present in the stranding area. Furthermore, given the continuous and ever-increasing nature of commercial shipping, it would seem unlikely that this activity could have been a factor in the 2005 UME without it also contributing to some other strandings at other (later) times during the period analysed.

Finally, although currents were not specifically included in the analyses in order to avoid extensive collinearity with tidal signals in the region concerned, it is highly likely that these will have played a role in determining the exact number of individuals that stranded [30]. However, it is also highly likely that the observed number of animals only represents a small fraction of the total involved in the UME, given the very low carcass recovery rate observed elsewhere [30].

#### Statistically tested factors

Of the statistically tested factors, we know that there was some navy presence during a period of relatively high lumpfish landings. Although both navy presence and lumpfish landings are correlated with higher stranding rates, neither the presence of the fishery or the navy was found to be positively correlated to the presence of fresh strandings. It is likely, therefore, that an unconsidered factor was also involved in this event. The need for the presence of multiple factors may explain why there have not been more similar stranding events at other times in the whole period analysed. For example, almost all other naval exercises in the period have been in the autumn when the lumpfish fishery is not present. Additionally, the sheer size of the LM05 exercise, in terms of the number of ships involved, might also have been important. The range of activities may have contributed as well. This NATO exercise reportedly involving 85 vessels (see S2), high-frequency sonar for mine clearing activities, both mid-frequency active sonar (MFAS) and low-frequency active sonar (LFAS: as per Norwegian response to the request for information, see Table S2) for anti-submarine activities, and live fire exercises. Despite the statistical correlation found in this analysis, it may be tempting to rule LM05 out as a contributing factor and conclude that some other untested factor is responsible. This would be due to the temporal resolution of the statistical model and the fact that the main exercise started several days after the first stranding and continued long after the strandings ended. However, naval activity must remain a possible contributing factor for the following reasons:

1. There were some confirmed pre-LM05 exercises involving various ships in various navies. Some of these did take place en-route through the stranding area. However the extent of any such pre-exercise testing and manoeuvres remains unknown, as do their exact dates and locations, because many navies were unable or unwilling to supply the necessary information upon request. These known activities and transits are likely to be only a small fraction of the total pre-LM05 manoeuvres.
2. A visual/acoustic survey of harbour porpoises in Danish waters just after the stranding period (a pilot study for SCANS II [63]) found unusually few harbour porpoises in the Kattegat and Skagerrak (SCANS II, unpubl. data). This finding may be consistent with an avoidance of the area after the initial exposure (as has been seen in beaked whales, Ziphiidae [17],[18]),
3. The most sensitive individuals in the population may have been quickly eliminated or displaced at the onset of activity, resulting in only a short period of strandings.

Furthermore, this is not the first time that navy sonar has been implicated, to some extent, in a stranding of harbour porpoises. In May 2003 the US naval vessel *USS SHOUP* used its MFAS in the eastern Strait of Juan de Fuca and Haro Strait between Vancouver Island (Canada) and San Juan Island (US) and, within a few days, 11 (confirmed) to 15 (reported) harbour porpoises stranded in the area [20]. A presumptive cause of death was determined for five of these individuals: “two cases of agonal or perimortem blunt force trauma, a single case of fibrinous peritonitis, one porpoise with salmonellosis, and one with a profound necrotizing pneumonia” ([20] page 53). However, as the animals were already in varying states of decomposition the investigations were limited, the possibility of acoustic trauma as a contributory factor in the mortality “could not be ruled out” ([20] page 55).

This highlights a substantial flaw in thinking at the time: that strandings related to sonar are caused by acoustic trauma. All evidence to date suggests that, at least in beaked whales, direct acoustic trauma from exposure to sonar is not necessary to cause strandings (see [56]). For example, it was calculated that received sound levels involved in the Bahamas 2000 beaked whale stranding were not even high enough to cause a temporary threshold shift in hearing [64]. Furthermore, as mentioned above, other species behave in a manner consistent with the reactions seen in beaked whales in response to exposure to unfamiliar or noxious sounds. Consequently, it is not appropriate to use a lack of acoustic trauma to determine that acoustic exposure was not involved in causing a stranding.

Thus, as a result of the statistical analysis in combination with the above-mentioned observations, but also with consideration of both the model limitations (see below) and the lack of detailed information on naval activities, we were unable to exclude naval activity as a one potential contributing element in what is likely to have been a combination of factors that ultimately led to the 2005 Danish porpoise UME.

One possible mechanism could be that the exposed porpoises may simply not have been paying attention to the fishing nets due to the presence of the noise from the naval activities. Dudok van Heel [65] first suggested that a lack of attention (e.g., due to focus elsewhere during to prey capture attempts) could lead to cetacean strandings. More recently, theoretical groundwork by Dukas [66] on the diversion of attention in general has been followed by empirical results in Caribbean hermit crabs (*Coenobita clypeatus*) [67],[68], three-spined sticklebacks (*Gasterosteus aculeatus*) [69] and possibly also greater mouse-eared bats (*Myotis myotis*) [70], demonstrating that disturbance from human activity can be distracting to an animal. The resulting redirection of part of an individuals' limited focus to the noise or disturbance then leaves it less able to detect prey, predators or potentially also the presence of nets.

### Model limitations

t is entirely possible (and actually appears quite likely) that something outside our analysis may be correlated with strandings and have contributed to the 2005 UME. For example, much of the variability in cod landings was not contained within the first 9 PCA axes, probably due to the fact that there is less variability over the year in the cod landings than is present in some of the other fisheries. As nets set for cod are the largest known causes of porpoise bycatch in Danish waters [35], this omission may explain why only weak correlations were found with independent variables in the final binomial model. Despite this, it was not possible to include more than 50% of the variability in the fish landings data as the model would have become too large and cumbersome.

Similarly, the spatial and temporal resolution of the model was limited by the fisheries and oceanographic data to ICES areas and 5-day periods respectively. As a consequence, it was not possible to consider any of the fine-scale features and events than may influence porpoise distributions and/or strandings (e.g., ebb and flow tides [71]).

Finally, despite the presence of legislation requiring that government agencies in most LM05 countries provide information when requested, several navies withheld various details of their activities. Due to this uncooperative nature, recognised national security issues and other limitations on information available on naval activities (e.g., time since the exercise), it was not possible to include the precise location or nature of the naval actions. Although a more complete data set of naval activity might have either strengthened or weakened the potential correlation between the number of fresh strandings and naval presence, national security concerns may render such details permanently unavailable to science. Moreover, we argue that the lack of detailed information on naval activities together with the lack of a consistent measure of freshness in the in-field strandings data precludes any further improvement in the spatial or temporal resolution of the analysis.

Despite the limitations, this study illustrates the potential for secondary, indirect impacts resulting from exposure of cetaceans to human disturbances. Specifically, the role of naval activity as a potential contributor to the 2005 harbour porpoise UME could not be ruled out. This further suggests that exposure to sonar, and potentially also other anthropogenic sounds, may lead to undetected mortalities through interactions with other human activities (i.e., cumulative impacts). In turn, this indicates that the widely-held belief in terrestrial environments that the discovery of a single carcass is indicative of more deaths elsewhere [72] can and should be applied to the marine environment as well. (This support is above and beyond that also offered recently in a study of carcass recovery rates [30].) Moreover, the idea that sound exposure could increase bycatch rates highlights the inappropriateness of the general focus on strandings in discussions of acoustic impacts on marine mammals [73]–[75]. In short, an exposed animal may die at sea and not strand, or instead may be bycaught and not identified as a casualty of the exposure. Thus, using identified strandings alone as a metric for the impact of an acoustic exposure will almost certainly underestimate the total impact of such exposure.

### General Conclusions

It is possible that exposure to naval sonar contributed to the 2005 Danish porpoise UME through a synergistic interaction with fisheries in Danish waters, resulting in increased bycatch. Considerable money and effort has been invested in attempts to reduce bycatch [76]–[79], which is a problem for many cetacean populations around the world [78],[80], including the Danish harbour porpoise [35]. Most efforts have focused on acoustic deterrent devices (ADDs) or “pingers”, which produce sounds that are supposed to deter cetaceans from approaching nets. However, these pingers also have a number of problems of their own, including improper use and maintenance, behavioural habituation (i.e., tolerance) by the animals and habitat exclusion [77],[81]–[84]. The results presented here hint at the fact that it may be possible to reduce (although probably not eliminate) bycatch by limiting other human activities that might distract the animals. Such a possibility definitely merits further research in this area.

Regardless of any specific conclusion, the findings in this study also clearly demonstrate that investigations of UMEs need to be more open to the possibility that one or more extraneous factors could be involved in leading to an otherwise apparently clear-cut cause of death. Additionally, a more standardised monitoring program for marine mammal strandings in Denmark is needed not only to react more quickly with specific investigations of fresh specimens during UMEs, but also to have a better overview on the current and developing health status of Danish populations. The discussion here also highlights the need for a wider appreciation for cumulative impacts in general, both scientifically and in terms of assessments underpinning management decisions.

## Supporting Information

Figure S1 **Variability of the 88 species-ICES area combinations captured in PC1 and PC2.** Biplot showing the relationship between the various species-ICES area combinations in the plane of PC1 and PC2 in grey. Species of particular interest in terms of prey or bycatch are highlighted and labelled in red, as are the species-ICES area combinations that have the largest proportion of their variability captured in PC1 (S Grey Gurnard), PC2 (S Catfish), PC3 (S Whiting), and PC4 (S Blue Ling) (see Table S3).
(TIF)

Table S1 **Summary of naval activity in Danish waters 2003–2008.** A summary of all the information obtained regarding military activity in Danish waters for the analysis period. All websites were accessed last on 12^th^ July 2012.
(DOCX)

Table S2 **Details of the Loyal Mariner 2005 exercise from enquiries.** A summary of the most pertinent information obtained from the navies of the various countries involved in the Loyal Mariner 2005 exercise regarding their activities. Note that it was not possible to confirm or refute temporal overlap in the stranding area for most navies based upon the lack of detail in the information provided. * = The column FOIA? denotes whether the particular country has (Y = yes) or has not (N = no) a Freedom of Information Act (FOIA), or similar legislation, designed to make most information on government activities available to the public upon request (with notable exceptions regarding national security issues and classified information). # = NATO has a policy to provide as much information as possible, but there is no regulatory requirement. Other notations: Y = Military hardware confirmed present through the information provided, although numbers were not provided; Prob = Military hardware likely to be present given the information provided; Maybe = Military hardware possibly present, although unclear given the information provided; U = Unknown: no information provided.
(DOCX)

Table S3 **Details of the principal component analysis.** Statistical details and components for each of the selected principal component analysis axes. Area-species catch data with loadings of ±0.5 or more in any particular axis are highlighted in bold and underlined. Species of particular interest in terms of prey or bycatch are highlighted in italics.
(DOCX)

## References

[1] Klinowska M (1988) Cetacean ‘navigation’ and the geomagnetic field. J Navigation 41: 52–71.

[2] Mignucci-Giannoni AA, Toyos-Gonzalez GM, Perez-Padilla J, Rodriguezlopez MA, Overing J (1999) Mass stranding of pygmy killer whales (*Feresa attenuata*) in the British Virgin Islands. J Mar Biol Assoc U.K 80: 759–760.

[3] Jauniaux T, Brosens L, Jacquinet E, Lambrigts D, Coignoul F (1997) Pathological investigations on sperm whales stranded on the Belgian and Dutch coasts. Biologie 67 Supplement 63–67.

[4] Lambertsen RH (1997) Natural disease problems of the sperm whales. Biologie 67 Supplement 105–112.

[5] Müller G, Siebert U, Wünschmann A, Artelt A, Baumgärtner W (2000) Immunohistochemical and serological investigation of morbillivirus infection in harbour porpoises (*Phocoena phocoena*) from the German Baltic and North Sea. Vet Microbiol 75: 17–25. 10865149 10.1016/s0378-1135(00)00209-1

[6] Siebert U, Wünschmann A, Weiss R, Frank H, Benke H, et al (2001) Post-mortem findings in harbour porpoises (*Phocoena phocoena*) from the German North and Baltic Seas. J Comp Pathol 124: 102–114. 11222006 10.1053/jcpa.2000.0436

[7] Bouquegneau JM, Debacker V, Gobert S, Nellissen JP (1997) Toxicological investigations on four sperm whales stranded on the Belgian coast: Inorganic contaminants. Biologie 67 Supplement 75–78.

[8] Jepson PD, Bennett PM, Deaville R, Allchin CR, Baker JR, et al (2005) Relationships between polychlorinated biphenyls and health status in harbor porpoises (*Phocoena phocoena*) stranded in the United Kingdom. Environ Toxicol Chem 24: 238–248. 15683190 10.1897/03-663.1

[9] Joiris CR, Holsbeek L, Bossicart M, Tapia G (1997) Mercury and organochlorines in four sperm whales stranded on the Belgian coast, November 1994. Biologie 67 Supplement 69–73.

[10] Siebert U, Joiris C, Holsbeek L, Benke H, Failing K, et al (1999) Potential relation between mercury concentrations and necropsy findings in cetaceans from German waters of the North and Baltic Seas. Mar Pollut Bull 38 (4) 285–295.

[11] Fernández A, Edwards JF, Rodriguez F, Espinosa de los Morteros A, Herraez P, et al (2005a) “Gas and fat embolic syndrome” involving a mass stranding of beaked whales (Family *Ziphiidae*) exposed to anthropogenic sonar signals. Vet Pathol 42: 446–457. 16006604 10.1354/vp.42-4-446

[12] Jepson PD, Arbelo M, Deaville R, Patterson IAP, Castro P, et al (2003) Gas bubble lesions in stranded cetaceans: was sonar responsible for a spate of whale deaths after an Atlantic military exercise? Nature 425: 575–576. 14534575 10.1038/425575a

[13] Jepson PD, Prahl S, Deaville R, Siebert U (2006) Postmortem Research Feasibility Study on Cetacean Ears. Final Report to the Department for Environment, Food and Rural Affairs 16 p. Available: http://randd.defra.gov.uk/Document.aspx?Document=WC04008_4190_FRP.pdf. Accessed 2012 Jul 18.

[14] Parsons ECM, Dolman SJ, Wright AJ, Rose NA, Burns WCG (2008) Navy sonar and cetaceans: Just how much does the gun need to smoke before we act? Mar Pollut Bull 56: 1248–1257. 18534632 10.1016/j.marpolbul.2008.04.025

[15] Evans DL, England GR (2001) Joint interim report Bahamas marine mammal stranding event 15–16 March 2000. Washington, D.C.: Department of the Navy and Department of Commerce, National Oceanic and Atmospheric Administration. 59 p.

[16] Cox TM, Ragen TJ, Read AJ, Vos E, Baird RW, et al (2006) Understanding the impacts of acoustic sound on beaked whales. J Cetacean Res Manage 7: 177–187.

[17] McCarthy E, Moretti D, Thomas L, Dimarzio N, Morrissey R, et al (2011) Changes in spatial and temporal distribution and vocal behavior of Blainville's beaked whales (*Mesoplodon densirostris*) during multiship exercises with mid-frequency sonar. Mar Mamm Sci 27 (3) E206–E226 doi:10.1111/j.1748-7692.2010.00457.x

[18] Tyack PL, Zimmer WMX, Moretti D, Southall BL, Claridge DE, et al (2011) Beaked whales respond to simulated and actual navy sonar. PLoS One 6 (3) e17009 doi:10.1371/journal.pone.0017009 21423729 10.1371/journal.pone.0017009 PMC3056662

[19] Tyack PL, Johnson M, Aguilar Soto N, Sturlese A, Madsen PT (2006) Extreme diving of beaked whales. J Exp Biol 209: 4238–4253. 17050839 10.1242/jeb.02505

[20] Norman SA, Raverty S, McLellan B, Pabst A, Ketten D, et al (2004) Multidisciplinary investigation of stranded harbor porpoises (*Phocoena phocoena*) in Washington State with an assessment of acoustic trauma as a contributory factor (2 May–2 June 2003). U.S. Dep. Commerce, NOAA Tech. Memo. NMFS-NWR-34 120.

[21] Jepson PD, Deaville R (compilers) (2009) Investigation of the common dolphin mass stranding event in Cornwall, 9th June 2008. Report to the Department for Environment, Food and Rural Affairs (under a variation to Contract CR0364). 30 p. Available: http://randd.defra.gov.uk/Document.aspx?Document=WC0601_8031_TRP.pdf. Accessed 2012 Jul 18.

[22] Breland K, Breland M (1966) Animal Behavior. New York: The Macmillan Company. 210 p.

[23] Wood FG (1979) The cetacean stranding phenomena: an hypothesis. In Geraci JR, St. Aubin DJ, editors. The biology of marine mammals: insights through strandings. Washington, D.C.: Marine Mammal Commission. pp. 129–188.

[24] Marcogliese DJ, Pietrock M (2011) Combined effects of parasites and contaminants on animal health: Parasites do matter. Trends Parasitol 27: 123–130. 21144800 10.1016/j.pt.2010.11.002

[25] Van Bressem MF, Raga JA, Di Guardo G, Jepson PD, Duignan PJ, et al (2009) Emerging infectious diseases in cetaceans worldwide and the possible role of environmental stressors. Dis Aquat Org 86: 143–157. 19902843 10.3354/dao02101

[26] Mazzariol S, Di Guardo G, Petrella A, Marsili L, Fossi CM, et al (2011) Sometimes Sperm Whales (Physeter macrocephalus) Cannot Find Their Way Back to the High Seas: A Multidisciplinary Study on a Mass Stranding. PLoS ONE 6 (5) e19417 doi:10.1371/journal.pone.0019417 21673789 10.1371/journal.pone.0019417 PMC3097202

[27] Vanselow KH, Ricklefs K (2005) Are solar activity and sperm whale *Physeter macrocephalus* strandings around the North Sea related? J Sea Res 53: 319–327.

[28] Vanselow KH, Ricklefs K, Colijn F (2009) Solar Driven Geomagnetic Anomalies and Sperm Whale (Physeter macrocephalus) Strandings Around the North Sea: An Analysis of Long Term Datasets. Open Mar Biol J 3: 89–94.

[29] Wright AJ (2005) Lunar cycles and sperm whales (*Physeter macrocephalus*) strandings on the North Atlantic coastlines of the British Isles and Eastern Canada. Mar Mamm Sci 21 (1) 145–149.

[30] Peltier H, Dabin W, Daniel P, Van Canneyt O, Dorémus G, et al (2012) The significance of stranding data as indicators of cetacean populations at sea: Modelling the drift of cetacean carcasses. Ecol Indic 18: 278–290.

[31] Nowacek DP, Johnson MP, Tyack PL (2004) North Atlantic right whales (*Eubalaena glacialis*) ignore ships but respond to alerting stimuli. Proc R Soc Lond B Biol Sci 271: 227–231. 10.1098/rspb.2003.2570 PMC1691586 15058431

[32] Todd S, Stevick P, Lien J, Marques F, Ketten D (1996) Behavioural effects to underwater explosions in Humpback Whales (*Megaptera novaeangliae*). Can J Zool 74: 1661–1672.

[33] Baird RW, Guenther TJ (1995) Account of harbour porpoise (Phocoena phocoena) strandings and bycatches along the coast of British Columbia. Rep Int Whal Comm (Spec. Issue) 16: 159–168.

[34] Siebert U, Gilles A, Lucke K, Ludwig M, Benke H, et al (2006) A decade of harbour porpoise occurrence in German waters: analyses of aerial surveys, incidental sightings and strandings. J Sea Res 56: 65–80.

[35] Vinther M, Larsen F (2004) Updated estimates of harbour porpoise (*Phocoena phocoena*) bycatch in the Danish North Sea bottom-set gillnet fishery. J Cetacean Res Manage 6: 19–24.

[36] Ross HM, Wilson B (1996) Violent Interactions between bottlenose dolphins and harbour porpoises. Proc R Soc Lond B Biol Sci 263 (1368) 283–286. 10.1098/rspb.1998.0414 PMC1689180 9699310

[37] Härkönen TB, Bäcklin M, Barrett T, Bergman A, Corteyn M, et al (2008) Mass mortality in harbour seals and harbour porpoises caused by an unknown pathogen. Vet Rec 162: 555–556. 18441352 10.1136/vr.162.17.555

[38] R Development Core Team (2011) R: A language and environment for statistical computing. Vienna, Austria: R Foundation for Statistical Computing. ISBN 3-900051-07-0: Available: http://www.R-project.org/. http://www.ambion.com/techlib/append/supp/tri.pdf. Accessed 2011 Oct 11.

[39] Kuiken M, García Hartmann M (1993) Proceedings of the First ECS Workshop on Cetacean pathology: Dissection Techniques and Tissue Sampling. European Cetacean Society Special Newsletter 17. Leiden, the Netherlands: European Cetacean Society. 39 p.

[40] Baker JR, Martin AR (1992) Causes of mortality and parasites and incidental lesions in harbour porpoises (*Phocoena phocoena*) from British waters. Vet Rec 130: 554–558. 1323164 10.1136/vr.130.25.554

[41] Siebert U, Weiss R (2009) Regional differences in bacteria flora in harbour porpoises from the North Atlantic: environmental effects. J Appl Microbiol 106: 329–337. 19120613 10.1111/j.1365-2672.2008.04006.x

[42] Weir CR, O'Brien SH (2000) Association of the harbour porpoise (*Phocoena phocoena*) with the western Irish sea front. In: Evans PGH, Pitt-Aiken R, Rogan E, editors. European Research on Cetaceans 14. Rome, Italy: European Cetacean Society. pp. 61–65.

[43] Sveegaard S, Teilmann J, Tougaard J, Dietz R, Mouritsen KN, et al (2011) High-density areas for harbor porpoises (*Phocoena phocoena*) identified by satellite tracking. Mar Mamm Sci 27 (1) 230–246.

[44] Maar M, Larsen J, Møller EF, Madsen KS, Wan Z, et al (2011) Ecosystem modelling across a salinity gradient from the North Sea to the Baltic Sea. Ecol Model 222: 1696–1711.

[45] Hathaway DH (2010) The Solar Cycle. Living Rev Solar Phys 7:1. Available: http://www.livingreviews.org/lrsp-2010-1. Accessed 2012 Oct 10.

[46] International Seismological Centre (2010) On-line Bulletin, Internatl Seis Cent, Thatcham, United Kingdom. Available: http://www.isc.ac.uk. Data Accessed: 2012 Oct 10.

[47] Ross A, Isaac S (2004) The Net Effect? A review of cetacean bycatch in pelagic trawls and other fisheries in the north-east Atlantic. A WDCS report for Greenpeace. Chippenham, U.K.: Whale and Dolphin Conservation Society U.K. 73 p.

[48] Jolliffe IT (2002) Principal Component Analysis (2nd ed). New York: Springer. pp. 487.

[49] Hildebrand JA (2005) Impacts of anthropogenic sound. In: Reynolds JE, Perrin WF,, Reeves RR, Montgomery S, Ragen TJ, editors. Marine mammal research: conservation beyond crisis. Baltimore, Maryland: The Johns Hopkins University Press. pp. 101–124.

[50] Nowacek DP, Thorne LH, Johnston DW, Tyack PL (2007) Responses of cetaceans to anthropogenic noise. Mamm Rev 37: 81–115.

[51] Southall BL, Bowles AE, Ellison WT, Finneran JJ, et al (2007) Marine mammal noise exposure criteria: initial scientific recommendations. Aquat Mamm 33: 411–522.

[52] Weilgart LS (2007) The impacts of anthropogenic ocean noise on cetaceans and implications for management. Can J Zool 85: 1091–1116.

[53] Nieukirk SL, Mellinger DK, Moore SE, Klinck K, Dziak RP, et al (2012) Sounds from airguns and fin whales recorded in the mid-Atlantic Ocean, 1999–2009. J Acoust Soc Am 131 (2) 1102–1112. 22352485 10.1121/1.3672648

[54] Urick RJ (1983) Principles of underwater sound (3rd ed.). NewYork: McGrawHill 444 p.

[55] O'Brien RM (2007) A caution regarding rules of thumb for variance inflation factors. Qual Quant 41: 673–690 DOI 10.1007/s11135-006-9018-6

[56] Hooker SK, Fahlman A, Moore MJ, Aguilar de Soto N, Bernaldo de Quirós Y, et al (2012) Deadly diving: the physiological and behavioural management of decompression stress in diving mammals. Proc R Soc Lond B Biol Sci 279: 1041–1050 doi:10.1098/rspb.2011.2088 10.1098/rspb.2011.2088 PMC3267154 22189402

[57] Sveegaard S, Andreasen H, Mouritsen KN, Jeppesen JP, Teilmann J, et al (2012) Correlation between the seasonal distribution of harbour porpoises and their prey in the Sound, Baltic Sea. Mar Biol doi:10.1007/s00227-012-1883-z

[58] Wünschmann A, Siebert U, Frese K, Weiss R, Lockyer C, et al (2001) Evidence of infectious diseases in harbour porpoises (*Phocoena phocoena*) hunted in the water of Greenland and by-caught in the German North Sea and Baltic Sea. Vet Rec 148: 715–720. 11430682 10.1136/vr.148.23.715

[59] Clausen B, Andersen S (1988) Evaluation of by-catch and health status of the harbour porpoise (*Phocoena phocoena*) in Danish waters. Danish Rev of Game Biol 13: 1–20.

[60] Jauniaux T, Petitjean D, Brenez C, Borrens M, Brosens L, et al (2002) Post-mortem findings and causes of death of harbour porpoises (*Phocoena phocoena*) stranded from 1990 to 2000 along the coastlines of Belgium and Northern France. J Comp Pathol 126: 243–253. 12056772 10.1053/jcpa.2001.0547

[61] Jepson PD, Baker JR, Kuiken T, Simpson VR, Kennedy S, et al (2000) Pulmonary pathology of harbour porpoises stranded in England and Wales between 1990 and 1996. Vet Rec 146: 721–728. 10901214 10.1136/vr.146.25.721

[62] Swenshon M, Lämmler C, Siebert U (1998) Identification and molecular characterization of beta-hemolytic Streptococci isolated from harbour porpoises (*Phocoena phocoena*) of the North and Baltic Sea. J Clin Microbiol 36: 1902–1906. 9650933 10.1128/jcm.36.7.1902-1906.1998 PMC104949

[63] SCANS II (2008) Small Cetaceans in the European Atlantic and North Sea (SCANS-II). Final report to the European Commission under project LIFE04NAT/GB/000245. Fife, Scotland, U.K.: University of St Andrews. 54 p + Appendicies. Available at http://biology.st-andrews.ac.uk/scans2/inner-finalReport.html. Accessed 2012 Jul 18.

[64] Hildebrand JA (2005) Introduction to acoustics. J Cetacean Res Manag 7 (Suppl.) 284–286.

[65] Dudok van Heel WH (1966) Navigation in Cetacea. P597–606. In: Norris KD, editor. Whales, dolphins and porpoises. Berkely and Los Angeles: University of California Press. 232 p.

[66] Dukas R (2004) Causes and consequences of limited attention. Brain Behav Evol 63: 197–210 doi:10.1159/000076781 15084813 10.1159/000076781

[67] Chan AAYH, Giraldo-Perez P, Smith S, Blumstein DT (2010) Anthropogenic noise affects risk assessment and attention: the distracted prey hypothesis. Biol Lett 6: 458–461 doi:10.1098/rsbl.2009.1081 20164080 10.1098/rsbl.2009.1081 PMC2936217

[68] Chan AAYH, Stahlman WD, Garlick D, Fast CD, Blumstein DT, et al (2010) Increased amplitude and duration of acoustic stimuli enhance distraction. Anim Behav 80: 1075–1079.

[69] Purser J, Radford AN (2011) Acoustic noise induces attention shifts and reduces foraging performance in three-spined sticklebacks (*Gasterosteus aculeatus*). PLoS ONE 6 (2) e17478 doi:10.1371/journal.pone.0017478 21386909 10.1371/journal.pone.0017478 PMC3046255

[70] Siemers BM, Schaub A (2010) Hunting at the highway: traffic noise reduces foraging efficiency in acoustic predators. Proc R Soc Lond B Biol Sci doi: 10.1098/rspb.2010.2262. 7 p 10.1098/rspb.2010.2262 PMC3081776 21084347

[71] Johnston DW, Westgate AJ, Read AJ (2005) Effects of fine scale oceanographic features on the distribution and movements of harbour porpoises (*Phocoena phocoena*) in the Bay of Fundy. Mar Ecol Prog Ser 295: 279–293.

[72] Wobeser G (1994) Investigation and management of disease in wild animals. New York: Plenum Press. 256 p.

[73] D'Amico A, Gisiner RC, Ketten DR, Hammock JA, Johnson C, et al (2009) Beaked Whale Strandings and Naval Exercises. Aquat Mamm 35: 452–472 doi:10.1578-AM.35.4.2009.452

[74] Filadelfo R, Mintz J, Michlovich E, D'Amico A, Tyack PL, et al (2009) Correlating Military Sonar Use with Beaked Whale Mass Strandings: What Do the Historical Data Show? Aquat Mamm 35: 435–444 doi:10.1578-AM.35.4.2009.435

[75] Filadelfo R, Pinelis YK, Davis S, Chase R, Mintz J, et al (2009) Correlating Whale Strandings with Navy Exercises off Southern California. Aquat Mamm 35: 445–451 doi:10.1578-AM.35.4.2009.445

[76] Buscaino G, Buffa G, Sarà G, Bellante A, Tonello Jr AJ, et al (2009) Pinger affects fish catch efficiency and damage to bottom gill nets related to bottlenose dolphins. Fish Sci 75: 537–544.

[77] Gönener S, Bilgin S (2009) The Effect of Pingers on Harbour Porpoise, *Phocoena phocoena* Bycatch and Fishing Effort in the Turbot Gill Net Fishery in the Turkish Black Sea Coast. Turkish J Fish Aquat Sci 9: 151–157.

[78] Hamer DJ, Childerhouse SJ, Gales NJ, (2010) Mitigating operational interactions between odontocetes and the longline fishing industry: a preliminary global review of the problem and of potential solutions. Report to the International Whaling Commission (IWC) Scientific Committee, Report no. SC/62/BC3. 30p.

[79] Orphanides CD, Wetmore S, Johnson A (2009) Update on Harbor Porpoise Take Reduction Plan Monitoring Initiatives: Compliance and Consequential Bycatch Rates from June 2007 through May 2008; Pinger Tester Development and Enforcement from January 2008 through July of 2009. Ref Doc. 09-14. Woods Hole, MA: US Dept Commer, Northeast Fish Sci Cent. 16 p. Available from: National Marine Fisheries Service, 166 Water Street, Woods Hole, MA 02543–1026.

[80] Read AJ, Drinker P, Northridge S (2006) Bycatch of Marine Mammals in U.S. and Global Fisheries. Conserv Biol 20 (1) 163–169. 16909669 10.1111/j.1523-1739.2006.00338.x

[81] Culik BM, Koschinski S, Tregenza N, Ellis GM (2001) Reactions of harbor porpoises *Phocoena phocoena* and herring *Clupea harengus* to acoustic alarms. Mar Ecol Prog Ser 211: 255–260.

[82] Franse R (2005) Effectiveness of acoustic deterrent devices (pingers). Leiden, the Netherlands: Universiteit Leiden, Centrum voor Milieuwetenschappen. 33 p.

[83] Haelters J, Camphuysen K (2009) The harbour porpoise in the southern North Sea: abundance, threats and research- & management proposals. Royal Belgian Institute of Natural Sciences (RBINS/MUMM) and the Royal Netherlands Institute for Sea Research (NIOZ); report commissioned by the International Fund for Animal Welfare (IFAW). 56 p.

[84] Teilmann J, Tougaard J, Miller LA, Kirketerp T, Hansen K, et al (2006) Reactions Of Captive Harbor Porpoises (*Phocoena Phocoena*) To Pinger-Like Sounds. Mar Mamm Sci 22 (2) 240–260.

