# Supplementary figures and images for: Possible Causes of a Harbour Porpoise Mass Stranding in Danish Waters in 2005
Source: PLoS One. 2013 Feb 27;8(2):e55553. doi: 10.1371/journal.pone.0055553 (PMC3584061; doi:10.1371/journal.pone.0055553)

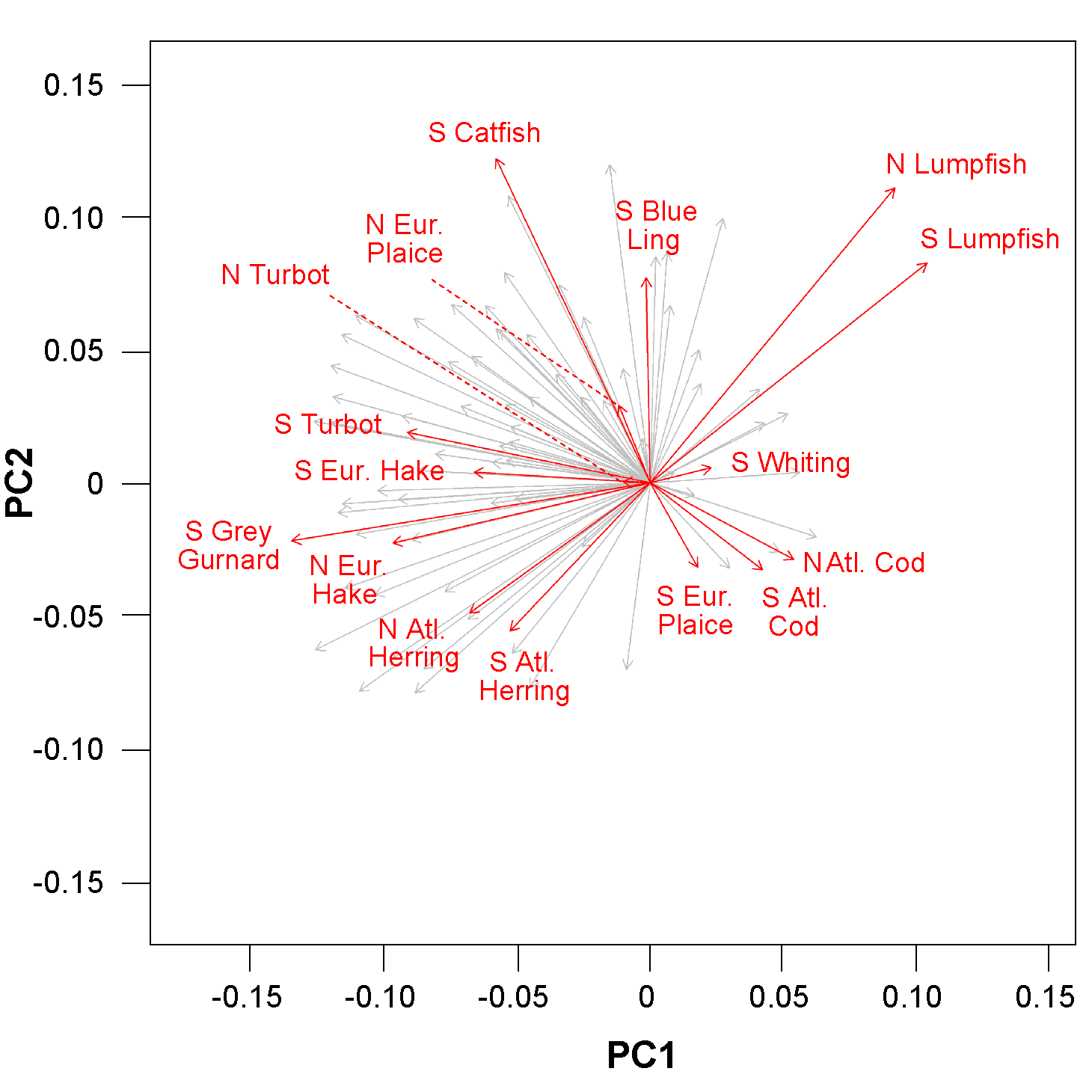

Supplement: Figure S1 — Variability of the 88 species-ICES area combinations captured in PC1 and PC2. Biplot showing the relationship between the various species-ICES area combinations in the plane of PC1 and PC2 in grey. Species of particular interest in terms of prey or bycatch are highlighted and labelled in red, as are the species-ICES area combinations that have the largest proportion of their variability captured in PC1 (S Grey Gurnard), PC2 (S Catfish), PC3 (S Whiting), and PC4 (S Blue Ling) (see Table S3). (TIF) [file pone.0055553.s001.tif]
