# Supplementary material for: Possible Causes of a Harbour Porpoise Mass Stranding in Danish Waters in 2005
Source: PLoS One. 2013 Feb 27;8(2):e55553. doi: 10.1371/journal.pone.0055553 (PMC3584061; doi:10.1371/journal.pone.0055553)
Supplement: Table S1 — Summary of naval activity in Danish waters 2003–2008. A summary of all the information obtained regarding military activity in Danish waters for the analysis period. All websites were accessed last on 12th July 2012. (DOCX) [file pone.0055553.s002.docx]

| **Exercise** | **Ships** | **Nations** | **Start** | **End** | **Location** | **Sonar activities?** | **Sources** |
| --- | --- | --- | --- | --- | --- | --- | --- |
| **DANEX 03** | 13+ | 7+ | 25 Jul 2003 | 5 Aug 2003 | At least in Kattegat and Skaggerak | Unknown, little information | Communications with the Danish Navy & http://www.mod.uk/NR/rdonlyres/EFB57FF1-0F80-44D1-9267-AB83A558292B/0/major_exercises04.pdf |
| **Blue Game 04** | 63 | 13 | 26 Apr 2004 | 14 May 2004 | “Denmark” | ASW & MCM | Communications with the Danish Navy & http://homepage.ntlworld.com/nigel.sadler1/page_article_Blue%20Game%202004.htm |
| **Loyal Mariner 05** | 85 | 18 | 11 Apr 2005 | 28 Apr 2005 | “Danish Waters” (Confirmed in Kateggat & Skaggerat) | ASW & MCM | Communcations with various navies & http://www.nato.int/docu/update/2005/04-april/e0411a.htm & http://forsvaret.dk/LoyalMariner05/eng/Pages/default.aspx |
| **DANEX 05** | 56 | 15 | 09 Sep 2005 | 23 Sep 2005 | “Danish Waters” (Kattegat through to the Baltic Sea confirmed) | ASW & MCM | Communications with the Danish Navy & http://forsvaret.dk/DANEX05/eng/Pages/default.aspx |
| **DANEX 06** | 35 | 7 | 01 Sep 2006 | 14 Sep 2006 | At least Skaggerak & Kattegat | Included “defense against other naval units” | Communications with the Danish Navy & http://forsvaret.dk/danex06/eng/Pages/default.aspx |
| **DANEX 07** | 20 | 5 | 22 Aug 2007 | 11 Sep 2007 | At least Skaggerat, Kateggat & Baltic Sea (Bornholm) | None known | Communications with the Danish Navy & http://forsvaret.dk/danex07/eng/Pages/default.aspx |
| **DANEX 08** | ? | 5 | 03 Sep 2008 | 16 Sep 2008 | At least Skaggerak & Kattegat | ASW & MCM | Communications with the Danish Navy & http://forsvaret.dk/danex08/eng/Pages/default.aspx |
