# Supplementary material for: Possible Causes of a Harbour Porpoise Mass Stranding in Danish Waters in 2005
Source: PLoS One. 2013 Feb 27;8(2):e55553. doi: 10.1371/journal.pone.0055553 (PMC3584061; doi:10.1371/journal.pone.0055553)
Supplement: Table S2 — Details of the Loyal Mariner 2005 exercise from enquiries. A summary of the most pertinent information obtained from the navies of the various countries involved in the Loyal Mariner 2005 exercise regarding their activities. Note that it was not possible to confirm or refute temporal overlap in the stranding area for most navies based upon the lack of detail in the information provided. * = The column FOIA? denotes whether the particular country has (Y = yes) or has not (N = no) a Freedom of Information Act (FOIA), or similar legislation, designed to make most information on government activities available to the public upon request (with notable exceptions regarding national security issues and classified information). # = NATO has a policy to provide as much information as possible, but there is no regulatory requirement. Other notations: Y = Military hardware confirmed present through the information provided, although numbers were not provided; Prob = Military hardware likely to be present given the information provided; Maybe = Military hardware possibly present, although unclear given the information provided; U = Unknown: no information provided. (DOCX) [file pone.0055553.s003.docx]

| **Country** | **FOIA? *** | **Ships** | **Subs** | **Sonar, etc?** | **LFA** | **MFA** | **Mine Hunting Sonar** | **Other Sound Sources** | **Earliest Arrival Date in Danish waters** | **Dates Involved in exercise** | **Comments** | **Activity coincident with stranding?** |
| --- | --- | --- | --- | --- | --- | --- | --- | --- | --- | --- | --- | --- |
| Belgium | Y | U | U | U | U | U | U | U | U | U | No response provided. | Not able to determine |
| Canada | Y | 3 | 0 | 1 | 0 | 0 | 1x 455 Hz? | 40/50 cal shot | 07 Apr 2005 at Frederikshavn, Denmark | 11-28 Apr 2005? (Conflicting information) | 20mm/50cal shooting on 5^th^ April in English Channel, other non-sonar exercises confirmed en-route on 7^th^ April. | Temporal overlap with ship presence confirmed |
| Denmark | Y | Y | U | Y | Maybe | Maybe | U | U | NA | All? | Any pre-exercises sonar usage would have been short duration & in Kattegat area. | Unknown |
| Estonia | Y | 1 | 0 | 1 | 0 | 0 | 1x 100/300 Hz | 0 | U | U | Very detailed response. Stated: Ship was harboured in Goteborg, Sweden. No activities within 200nm of coast of Jutland. | No |
| Finland | Y | 1 | 0 | U | 0 | 0 | U | 0 | U | U | Stated: no activities within 200nm of coast of Jutland. | Not able to determine |
| France | Y | U | U | U | U | U | U | U | U | U | No specific information provided, only on general environmental procedures. | Not able to determine |
| Germany | Y | U | U | U | U | U | U | U | U | U | Stated: LM05 involved: MFA ASW, minesweeping, and detonations. No details specific to Germany provided. | Not able to determine |
| Iceland | Y | 0 | 0 | 0 | 0 | 0 | 0 | 0 | NA | NA | No naval involvement. | No |
| Latvia | Y | U | U | U | U | U | U | U | U | U | No response provided. | Not able to determine |
| Lithuania | N | 1 | 0 | Y | 0 | 0 | 1x 100/300 Hz | 0 | 13 Apr 2005 at exercise | 13-27 Apr 2005 |  | No |
| NATO | Policy Only # | - | - | - | - | - | - | - | - | - | No response to formal information request. Informal inquiries yielded information that ran contrary to website information, which was later stated to be more reliable by NATO personnel. | - |
| Netherlands | Y | U | U | U | U | U | U | U | U | U | No information provided. | Not able to determine |
| Norway | Y | 6 | 0 | Y | 1 | 0 | 4x 210-220 Hz | No record | U | From 12 Apr |  | Not able to determine |
| Poland | Y | 2 | 1 | Prob | U | U | 1+ | U | U | U | 1 mine-hunter confirmed | Not able to determine |
| Spain | N | U | U | U | U | U | U | U | U | U | No response provided. | Not able to determine |
| Sweden | Y | U | U | U | U | U | U | U | U | U | No response provided. | Not able to determine |
| Ukraine | N | 0 | 0 | 0 | 0 | 0 | 0 | 0 | NA | NA | No naval involvement, marines only. | No |
| UK | Y | 6 | 0 | Y | 0 | 0 | 6x 100-300 Hz | At very least, weapons fired. | 07 Apr 2011 within 100km of the Stranding location | min 13-29 Apr 2005 | Detailed response provided. 1 ship confirmed on action stations (from 9:30am local time) conduction training exercises in stranding area on 7th April with at least 1 other vessel. This vessel had left Portsmouth with 1 other UK and 2 Canadian ships on 5^th^ April. | Temporal overlap with ship presence confirmed |
| USA | Y | 1+ | 1+ | Prob | U | U | U | U | U | U | Almost no information provided, despite official FOIA request. At least 1 high speed vessel & 1 sub involved. | Not able to determine |
