# Supplementary material for: Possible Causes of a Harbour Porpoise Mass Stranding in Danish Waters in 2005
Source: PLoS One. 2013 Feb 27;8(2):e55553. doi: 10.1371/journal.pone.0055553 (PMC3584061; doi:10.1371/journal.pone.0055553)
Supplement: Table S3 — Details of the principal component analysis. Statistical details and components for each of the selected principal component analysis axes. Area-species catch data with loadings of ±0.5 or more in any particular axis are highlighted in bold and underlined. Species of particular interest in terms of prey or bycatch are highlighted in italics. (DOCX) [file pone.0055553.s004.docx]

|  | PC1 | PC2 | PC3 | PC4 | PC5 | PC6 | PC7 | PC8 | PC9 |
| --- | --- | --- | --- | --- | --- | --- | --- | --- | --- |
| Standard deviation | 4.131 | 2.867 | 2.223 | 2.013 | 1.544 | 1.417 | 1.386 | 1.352 | 1.296 |
| Proportion of Variance | 0.194 | 0.093 | 0.056 | 0.046 | 0.027 | 0.023 | 0.022 | 0.021 | 0.019 |
| Cumulative Proportion | 0.194 | 0.287 | 0.343 | 0.389 | 0.417 | 0.439 | 0.461 | 0.482 | 0.501 |
| ***S Lumpfish*** | ***0.647*** | ***0.499*** | ***-0.180*** | ***0.243*** | ***-0.100*** | ***0.039*** | ***-0.028*** | ***0.038*** | ***-0.155*** |
| ***N Lumpfish*** | ***0.575*** | ***0.666*** | ***-0.091*** | ***0.143*** | ***-0.027*** | ***0.091*** | ***0.046*** | ***0.021*** | ***-0.066*** |
| N Common Dab | 0.391 | -0.118 | -0.135 | 0.023 | -0.147 | -0.186 | -0.055 | -0.301 | 0.209 |
| N Haddock | 0.349 | 0.024 | -0.042 | 0.324 | -0.011 | -0.035 | 0.125 | -0.179 | 0.103 |
| ***N Atlantic Cod*** | ***0.328*** | ***-0.169*** | ***0.163*** | ***-0.111*** | ***0.121*** | ***-0.115*** | ***0.088*** | ***0.035*** | ***-0.047*** |
| S Common Dab | 0.327 | 0.155 | -0.028 | 0.124 | -0.066 | -0.002 | -0.160 | -0.184 | 0.275 |
| S Saithe | 0.300 | -0.155 | -0.018 | -0.020 | 0.076 | 0.126 | -0.114 | -0.238 | -0.111 |
| *S Atlantic Cod* | *0.270* | *-0.197* | *0.129* | *-0.014* | *0.261* | *0.070* | *-0.060* | *0.108* | *0.196* |
| S Haddock | 0.268 | 0.138 | -0.012 | 0.048 | 0.094 | 0.069 | 0.287 | -0.445 | -0.091 |
| N Norway Lobster | 0.253 | 0.213 | -0.166 | 0.070 | 0.096 | 0.023 | -0.113 | -0.056 | 0.086 |
| S Norway Lobster | 0.190 | -0.196 | -0.092 | -0.103 | -0.046 | -0.120 | -0.040 | -0.353 | 0.057 |
| S Rays Skates | 0.167 | **0.598** | -0.303 | -0.360 | 0.110 | -0.160 | 0.002 | 0.008 | 0.047 |
| S Whiting | 0.147 | 0.032 | **-0.680** | 0.218 | -0.164 | -0.167 | -0.212 | 0.205 | -0.019 |
| S Rabbitfish | 0.123 | 0.224 | -0.131 | 0.078 | 0.188 | 0.079 | **0.539** | -0.349 | -0.008 |
| N North Deepwater Prawn | 0.106 | 0.302 | -0.111 | -0.102 | -0.135 | 0.238 | -0.346 | 0.024 | 0.033 |
| N Lemon Sole | 0.103 | -0.029 | -0.283 | 0.186 | 0.044 | -0.208 | -0.190 | -0.384 | -0.027 |
| ***S European Plaice*** | ***0.102*** | ***-0.193*** | ***0.124*** | ***-0.100*** | ***0.035*** | ***0.045*** | ***0.032*** | ***0.108*** | ***0.064*** |
| S Lemon Sole | 0.063 | 0.020 | -0.264 | 0.224 | -0.106 | 0.300 | -0.211 | -0.194 | 0.056 |
| S Greenland Halibut | 0.053 | 0.399 | 0.268 | 0.314 | 0.056 | 0.199 | 0.163 | 0.149 | -0.048 |
| S Tusk | 0.033 | **0.524** | -0.344 | -0.438 | 0.161 | -0.111 | 0.079 | -0.010 | 0.209 |
| S Roundnose Grenadier | 0.011 | **0.511** | -0.316 | **-0.546** | 0.251 | -0.170 | 0.115 | -0.028 | 0.043 |
| S Blue Ling | -0.014 | 0.464 | -0.236 | **-0.619** | 0.085 | -0.117 | 0.107 | -0.029 | -0.064 |
| S North Deepwater Prawn | -0.020 | -0.016 | -0.001 | -0.019 | 0.025 | 0.209 | -0.077 | 0.009 | 0.067 |
| N Whelk | -0.025 | 0.097 | 0.050 | -0.059 | -0.079 | 0.428 | -0.178 | -0.044 | 0.116 |
| N Common Shrimp | -0.049 | 0.260 | -0.092 | 0.311 | -0.073 | -0.120 | -0.093 | 0.081 | -0.062 |
| S Witch Flounder | -0.056 | -0.420 | 0.180 | 0.086 | -0.018 | 0.042 | -0.016 | -0.305 | -0.018 |
| ***N Turbot*** | ***-0.061*** | ***0.007*** | ***-0.342*** | ***0.058*** | ***-0.034*** | ***0.103*** | ***-0.078*** | ***-0.292*** | ***0.245*** |
| ***N European Plaice*** | ***-0.084*** | ***0.179*** | ***-0.048*** | ***-0.099*** | ***-0.074*** | ***0.181*** | ***0.280*** | ***0.201*** | ***-0.017*** |
| S Atlantic Halibut | -0.095 | **0.720** | -0.062 | 0.339 | -0.092 | 0.050 | 0.107 | -0.105 | 0.022 |
| N Whelk Periwinkles | -0.105 | 0.188 | -0.090 | -0.176 | 0.282 | 0.328 | -0.081 | -0.147 | -0.392 |
| S Common Sole | -0.156 | 0.374 | -0.137 | 0.306 | 0.166 | -0.023 | -0.092 | -0.087 | -0.274 |
| N Common Sole | -0.164 | -0.144 | -0.310 | -0.201 | -0.141 | 0.071 | -0.057 | 0.071 | -0.064 |
| N Rays Skates | -0.170 | 0.195 | -0.096 | 0.013 | 0.292 | 0.182 | 0.120 | 0.043 | 0.225 |
| N European Flounder | -0.212 | 0.453 | 0.145 | 0.147 | 0.113 | 0.016 | -0.022 | 0.017 | -0.121 |
| N Saithe | -0.216 | 0.245 | 0.011 | 0.055 | -0.074 | 0.041 | 0.161 | -0.267 | 0.048 |
| N Megrim | -0.285 | 0.200 | 0.163 | -0.234 | -0.039 | -0.065 | -0.291 | -0.002 | 0.034 |
| N Pollan | -0.286 | 0.338 | 0.079 | -0.330 | -0.272 | 0.313 | -0.149 | -0.156 | -0.099 |
| N Sprat | -0.292 | -0.465 | -0.450 | -0.277 | -0.013 | 0.089 | 0.319 | -0.019 | 0.034 |
| S Greater Weever | -0.315 | -0.387 | -0.042 | 0.028 | -0.143 | -0.299 | 0.236 | 0.115 | -0.019 |
| S Sprat | -0.317 | -0.036 | -0.367 | -0.302 | 0.222 | 0.179 | 0.107 | -0.010 | -0.218 |
| N Monk | -0.328 | 0.126 | -0.136 | 0.065 | -0.025 | 0.033 | 0.010 | -0.001 | 0.199 |
| S Garfish | -0.328 | 0.093 | 0.240 | -0.336 | 0.070 | -0.205 | 0.046 | 0.108 | 0.308 |
| S Ling | -0.330 | **0.648** | -0.177 | 0.073 | -0.210 | -0.066 | 0.072 | -0.055 | 0.014 |
| ***S Atlantic Herring*** | ***-0.334*** | ***-0.336*** | ***-0.345*** | ***-0.167*** | ***-0.147*** | ***0.180*** | ***0.085*** | ***0.115*** | ***-0.092*** |
| N Witch Flounder | -0.338 | 0.049 | -0.225 | 0.070 | -0.165 | 0.052 | 0.026 | -0.024 | 0.437 |
| N Sandeel | -0.339 | 0.477 | 0.431 | -0.205 | 0.090 | -0.002 | -0.183 | 0.067 | -0.017 |
| N Tusk | -0.342 | 0.180 | -0.220 | -0.119 | 0.290 | 0.064 | 0.010 | 0.022 | 0.120 |
| S Picked Dogfish | -0.346 | 0.346 | -0.235 | 0.065 | 0.406 | -0.187 | 0.028 | -0.052 | 0.067 |
| S Monk | -0.354 | 0.083 | -0.083 | 0.135 | -0.172 | 0.157 | -0.046 | -0.218 | -0.058 |
| S Sandeel | -0.360 | 0.349 | 0.459 | 0.010 | 0.221 | -0.047 | -0.159 | 0.099 | -0.182 |
| S Catfish | -0.360 | **0.735** | 0.284 | -0.008 | -0.274 | -0.063 | 0.068 | -0.089 | -0.003 |
| N Atlantic Horsemackerel | -0.378 | 0.046 | -0.025 | -0.337 | -0.159 | -0.061 | -0.138 | -0.089 | -0.041 |
| S Blue Whiting | -0.378 | -0.046 | -0.375 | **-0.556** | -0.110 | 0.068 | -0.113 | 0.020 | -0.150 |
| S European Flounder | -0.390 | 0.400 | -0.204 | 0.052 | -0.173 | -0.021 | 0.128 | 0.060 | -0.338 |
| ***S European Hake*** | ***-0.407*** | ***0.023*** | ***-0.120*** | ***0.146*** | ***0.316*** | ***-0.047*** | ***-0.335*** | ***-0.218*** | ***0.119*** |
| S Pollack | -0.413 | 0.285 | -0.076 | 0.244 | -0.397 | 0.019 | 0.144 | -0.007 | 0.073 |
| ***N Atlantic Herring*** | ***-0.421*** | ***-0.295*** | ***-0.264*** | ***0.075*** | ***0.099*** | ***0.286*** | ***0.086*** | ***0.059*** | ***-0.080*** |
| N Blue Whiting | -0.425 | -0.309 | -0.390 | -0.133 | -0.054 | 0.269 | 0.076 | -0.005 | -0.064 |
| N Whiting | -0.448 | 0.172 | -0.338 | -0.025 | 0.057 | 0.127 | -0.290 | 0.235 | 0.005 |
| S Unknown Species | -0.462 | 0.404 | -0.271 | -0.122 | -0.123 | -0.243 | -0.175 | 0.042 | 0.125 |
| N Atlantic Halibut | -0.470 | 0.274 | **0.537** | 0.209 | -0.035 | 0.087 | 0.186 | 0.204 | 0.124 |
| N Porbeagle | -0.484 | -0.244 | -0.275 | -0.160 | -0.027 | -0.089 | 0.149 | -0.036 | 0.053 |
| S Brill | **-0.501** | 0.063 | -0.327 | 0.463 | 0.201 | -0.133 | 0.165 | 0.008 | -0.211 |
| S Cuttlefish | **-0.527** | -0.426 | -0.329 | 0.230 | 0.042 | -0.110 | -0.042 | 0.080 | 0.111 |
| N Surmullet | **-0.546** | 0.029 | 0.481 | -0.228 | 0.047 | -0.208 | 0.127 | -0.182 | 0.122 |
| N Cuttlefish | **-0.552** | -0.477 | -0.344 | 0.126 | 0.192 | 0.106 | -0.016 | 0.198 | 0.121 |
| N Brill | **-0.557** | 0.371 | -0.018 | 0.222 | 0.116 | 0.319 | 0.150 | 0.111 | 0.188 |
| S Mullets | **-0.559** | -0.129 | 0.166 | 0.168 | 0.156 | 0.116 | -0.173 | -0.140 | -0.173 |
| ***S Turbot*** | ***-0.569*** | ***0.110*** | ***-0.261*** | ***0.373*** | ***-0.052*** | ***-0.189*** | ***0.007*** | ***-0.005*** | ***-0.111*** |
| N Pollack | **-0.585** | 0.156 | -0.225 | 0.129 | -0.310 | -0.028 | 0.102 | -0.086 | 0.013 |
| N Picked Dogfish | **-0.586** | -0.038 | -0.007 | 0.157 | 0.458 | -0.019 | 0.101 | 0.074 | 0.028 |
| N Tope | **-0.602** | -0.396 | -0.190 | -0.030 | 0.052 | -0.068 | 0.007 | -0.123 | -0.078 |
| ***N European Hake*** | ***-0.605*** | ***-0.136*** | ***0.052*** | ***0.271*** | ***0.347*** | ***-0.019*** | ***-0.244*** | ***-0.103*** | ***0.047*** |
| S European Lobster | **-0.638** | -0.015 | 0.137 | -0.247 | -0.248 | -0.167 | -0.058 | -0.042 | -0.144 |
| S European Eel | **-0.648** | -0.254 | 0.167 | 0.015 | 0.129 | 0.020 | -0.031 | -0.118 | 0.106 |
| S Tub Gurnard | **-0.679** | -0.472 | 0.113 | 0.113 | -0.225 | -0.062 | 0.046 | 0.012 | -0.053 |
| N Mullets | **-0.689** | -0.117 | 0.235 | -0.124 | 0.145 | -0.021 | -0.062 | -0.169 | -0.025 |
| N Marine Crabs | **-0.690** | 0.377 | 0.036 | 0.055 | 0.092 | -0.057 | 0.047 | 0.040 | 0.002 |
| S Atlantic Mackerel | **-0.722** | -0.234 | 0.189 | -0.107 | -0.140 | 0.026 | -0.084 | -0.067 | -0.024 |
| N Catfish | **-0.724** | 0.333 | 0.272 | 0.012 | -0.049 | 0.147 | 0.020 | -0.010 | 0.170 |
| N European Lobster | **-0.726** | -0.042 | 0.033 | -0.085 | 0.004 | -0.194 | -0.046 | -0.111 | -0.165 |
| N Atlantic Mackerel | **-0.739** | -0.065 | 0.359 | -0.196 | -0.053 | 0.008 | -0.053 | -0.128 | -0.019 |
| N Unknown Species | **-0.743** | 0.194 | -0.027 | 0.030 | -0.040 | 0.108 | -0.030 | 0.065 | 0.205 |
| S Marine Crabs | **-0.749** | 0.262 | -0.021 | 0.022 | 0.063 | -0.243 | -0.124 | 0.061 | -0.162 |
| N Grey Gurnard | **-0.760** | 0.135 | 0.072 | -0.205 | -0.018 | 0.190 | 0.041 | -0.083 | 0.053 |
| N Tub Gurnard | **-0.785** | -0.379 | 0.145 | 0.071 | -0.006 | 0.140 | 0.073 | 0.011 | 0.014 |
| N Ling | **-0.787** | 0.140 | -0.169 | 0.093 | -0.113 | 0.094 | -0.011 | 0.027 | 0.146 |
| S Grey Gurnard | **-0.840** | -0.130 | 0.045 | 0.071 | -0.189 | -0.097 | 0.130 | 0.005 | -0.070 |
